# Flavonol glycoside complanatoside A requires FOXO/DAF-16, NRF2/SKN-1, and HSF-1 to improve stress resistances and extend the life span of *Caenorhabditis elegans*

**DOI:** 10.3389/fphar.2022.931886

**Published:** 2022-08-22

**Authors:** Lin Tan, Zhuo-Ya Zheng, Lv Huang, Zhong Jin, Su-Lian Li, Gui-Sheng Wu, Huai-Rong Luo

**Affiliations:** ^1^ Key Laboratory for Aging and Regenerative Medicine, Department of Pharmacology School of Pharmacy, Southwest Medical University, Luzhou, Sichuan, China; ^2^ Department of Pharmacy, Guang’an People’s Hospital, Guang’an, Sichuan, China; ^3^ Luzhou City Hospital of Traditional Chinese Medicine, Luzhou, Sichuan, China; ^4^ Affiliated Traditional Chinese Medicine Hospital of Southwest Medical University, Luzhou, Sichuan, China; ^5^ Central Nervous System Drug Key Laboratory of Sichuan Province, Luzhou, Sichuan, China; ^6^ Key Laboratory of Medical Electrophysiology, Ministry of Education and Medical Electrophysiological Key Laboratory of Sichuan, Institute of Cardiovascular Research, Southwest Medical University, Luzhou, Sichuan, China

**Keywords:** *Caenorhabditis elegans*, aging, complanatoside A, DAF-16/FOXO, neurodegenerative diseases, oxidative stress, proteostasis

## Abstract

Aging is associated with the increased risk of most age-related diseases in humans. Complanatoside A (CA) is a flavonoid compound isolated from the herbal medicine Semen Astragali Complanati. CA was reported to have potential anti-inflammatory and anti-oxidative activities. In this study, we investigated whether CA could increase the stress resistance capability and life span of *Caenorhabditis elegans*. Our results showed that CA could extend the longevity of *C. elegans* in a dosage-dependent manner, while 50 μM of CA has the best effect and increased the life span of *C. elegans* by about 16.87%. CA also improved the physiological functions in aging worms, such as enhanced locomotor capacity, and reduced the accumulation of the aging pigment. CA could also reduce the accumulation of toxic proteins (α-synuclein and β-amyloid) and delay the onset of neurodegenerative disorders, such as Alzheimer’s disease and Parkinson’s disease, in models of *C. elegans*. Further investigation has revealed that CA requires DAF-16/FOXO, SKN-1, and HSF-1 to extend the life span of *C. elegans*. CA could increase the antioxidation and detoxification activities regulated by transcription factor SKN-1 and the heat resistance by activating HSF-1 that mediated the expression of the chaperone heat shock proteins. Our results suggest that CA is a potential antiaging agent worth further research for its pharmacological mechanism and development for pharmaceutical applications.

## Introduction

Aging is considered a major risk factor for many diseases, such as cancer, diabetes, high blood pressure, and Alzheimer’s and Parkinson’s diseases (Santoro et al., 2020). The growing aged population has become a major global problem and substantial challenge for healthcare systems (Galkin et al., 2020). Therefore, there is an urgent need to understand the basic molecular mechanisms of aging and to find ways to reverse (or at least delay) the onset of chronic age-related neurodegenerative diseases. Natural compounds are one of the main sources for developing potential drugs to intervene the aging process. Notably, some flavonoids, such as quercetin, catechin, curcumin, and green tea polyphenols, have been shown to prolong life span and delay the onset of age-related diseases (Falcone Ferreyra et al., 2012; Zheng et al., 2017).

Semen Astragali Complanati is the dry and mature seed of *Astragalus complanatus R. Br.* (Ng et al., 2014). Modern pharmacological research studies have demonstrated that Semen Astragali Complanati has antipyretic, anti-inflammatory, and analgesic properties (Ng et al., 2014; Gao et al., 2019). Flavonoids from Semen Astragali Complanati could improve the activity of superoxidase dismutase (SOD), reduce the level of malondialdehyde (MDA) and nitric oxide (NO), and enhance the nonspecific immune function in the aging mice model (Qi et al., 2011). The total flavonoids of *A. complanatus* has the ability to scavenge DDPH (2,2-diphenyl-1-picrylhydrazyl) oxygen free radicals and has an antiaging effect in *Drosophila melanogaster* (Li et al., 2016). The flavonoid complanatoside A (CA) is the main bioactive part of Semen Astragali Complanati (Qi et al., 2011). Studies have found that it can reduce cholesterol, triglyceride, peripheral resistance, blood pressure, and serum alanine aminotransferase, increase liver glycogen, and protect the liver of model animals (Meng, 2011). However, no further studies have been conducted on the antiaging mechanism of monomer compounds of Semen Astragali Complanati.

*C. elegans*, as a classical model organism for the study of aging, has a transparent and shorter life span, clear genetic background, and conservative pathways regulating cellular protection and aging (Acharya et al., 2006). Dietary restriction (DR) can extend the life span and delay the emergence of age-related diseases in model organisms and humans (Riera et al., 2016). DR leads to reprogramming of mitochondrial energy metabolism and inhibition of anabolic processes, activation of AMPK, decreased insulin/insulin-like growth factor (IGF)-1 signaling (IIS) and TOR signaling (Riera et al., 2016). IIS is also evolutionarily conserved in *Drosophila* and mice, and probably, in humans (Fontana et al., 2010). In this pathway, the ligand binding to DAF-2/InR activates its tyrosine kinase activity and initiates a cascade of sequential phosphorylation of kinases: AGE-1/PI3K, PDK-1, AKT-1/2, and SGK-1 (Paradis and Ruvkun, 1998). Then, AKT and SGK-1 phosphorylate and inactivate the FOXO transcription factor DAF-16 by preventing its translocation to the nucleus. In long-lived *daf-2* mutants, reduced IIS not only activates DAF-16 but also activates HSF-1 and SKN-1, leading to an increase in stress resistance of the *daf-2* mutant. TOR is a major cellular nutrient-sensing pathway that responds to nutrients, growth factors, and cellular energy status. TOR is a part of two structurally and functionally distinct complexes, TORC1 and TORC2. The downstream effectors of TORC1 include the ribosomal protein S6 kinase 1 (S6K1) and the eukaryotic translation initiation factor 4E-binding protein (4E-BP) (Blackwell et al., 2019).

Mild disruption of the function of different ETC subunits (such as *atp*-*3*, *isp*-*1*, *cco*-*1*, *nuo*-*2*, and *frh*-*1*) by mutation or RNAi inhibition could induce the effect of mitohormesis to promote *C*. *elegans* longevity (Blackwell et al., 2019). Increased life span is usually accompanied by higher resistance to adverse environmental stressors.

In this study, we used *C. elegans* as the model organism to investigate anti-stress and antiaging activities of CA.

## Methods

### Materials

The flavonoid CA was supplied by Shanghai Yuanye Bio-Technology Co. Ltd. (Shanghai, China, purity ≥95%). The nematode growth medium (NGM) was prepared from NaCl, peptone, agar, cholesterol, CaCl_2_, and MgSO_4_. The nematode lysis reagent was prepared from NaOH and NaClO. The nematode anesthetic (−)-tetramisole hydrochloride, paraquat, Oil Red O, and the fluorescent probe 2′,7′-dichlorodihydrofluorescein diacetate (H2DCF-DA) were purchased from Sigma-Aldrich (St. Louis, MO, United States).

### Worm strains and maintenance

All worm strains were provided by the Caenorhabditis Genetics Center (CGC, University of Minnesota, Minneapolis, MN). Except for special cases, worms were cultured on a medium with *E. coli* OP50 in an incubator with constant temperature (20°C) and humidity (60%). The nematode strains were as follows: N2 (Bristol, wild-type), RB759 *akt-1*(*ok525*)*V*, VC204 *akt-2*(*ok393*)*X*, CB1370 *daf-2*(*e1370*) *III*, CF1038 *daf-16* (*mu86*) *I*, EU1 *skn-1*(*zu67*)*IV*, PS3551 *hsf-1*(*sy441*)*I*, MQ887 *isp-1*(*qm150*) *IV*, TK22 *mev-1*(*kn1*)*III*, CB4876 *clk-1*(*e2519*)*III*, RB754 *aak-2*(*ok524*)*X*, DA1116 *eat-2*(*ad1116*) *II*, CF1903 *glp-1*(*e2144*) *III*, AA89 *daf-12* (*rh274*), BX165 *nhr-80* (*tm1011*), TJ356 *zIs356* [daf-16p *::* daf-16a */b::GFP +* rol-6(su1006)], LD1 *ldIs7* [skn-1b */c::GFP +* rol-6(su1006)], CF1553 *muIs84* [(*pAD76*) sod-3p *::GFP +* rol-6(su1006)], CL2166 *dvIs19* [(*pAF15*)gst-4p *::GFP::NLS*] *III*, SJ4058 *zcIs9* [hsp-60 *::GFP +* lin-15(*+*)], SJ4100 *zcIs13* [hsp-6 *::GFP*], SJ4005 *zcIs4* [hsp-4 *::GFP*] *V*, CL4176 *dvIs27* [myo-3p *::A-Beta* (*1–42*)*::* let-851 *3′UTR*) *+* rol-6(su1006)] *X*, CL2006 *dvIs2* [*pCL12*(unc-54 */human Abeta peptide 1–42 minigene*) *+* rol-6(su1006)], NL5901 *pkIs2386* [unc-54p *::alphasynuclein::YFP +* unc-119(*+*)], and BZ555 *egIs1* [dat-1p *::GFP*]*.*

### Life span assays

Life span analysis was performed at 20°C unless otherwise stated. Late L4 larva or early adults were transferred to NGM plates containing inactivated OP50 (65°C for 30 min) and 20 μM of FUdR to inhibit the growth of progeny. The day that L4 larvae or young adults were transferred to an NGM plate for life span assay was defined as test day 0. Every other day, the animals were moved to fresh plates with or without CA to avoid generational confusion. Live animals were scored at the same time (Fire et al., 1998). Worms those failed to respond to mechanical stimuli were considered dead. If the worms crawled out of the Petri dish, showing compressed internal organs, or died from hatching their offspring in the womb, they were removed from statistics. Life span assays were repeated three times with 60 worms per assay.

### Stress resistance assay

For the heat stress resistance test, the synchronized larvae were spread on the NGM plates, cultured in an incubator at 20°C, and then transferred to NGM plates with or without CA at the late L4 stage or early adult stage. The worms were transferred to 35°C on the 10th day of adulthood. The death of the worm was observed and counted every 2 h.

For the anti-oxidative stress test, pretreatment was the same as the heat stress resistance test: on the 10th day of adulthood, the worms were transferred to the NGM plates containing 20 mM of paraquat. The death of the worms was observed and counted every other day.

All statistics and analysis methods of stress assays were the same as the life span experiment. The sample size of each experiment was at least 60 worms. Each stress assay contained at least three independently repeated experiments.

### Body bending behavior test

In the body bending experiment, the initial treatment was the same as the life span test. The nematode was lifted into water drops and stabilized for 1 min on the 5th and 10th day of adulthood. After that, the number of body wobbling was recorded under the microscope for 20 s, and the back and forth swing of the tail was calculated as one bending movement.

### Determination of reactive oxygen species

Synchronized L1 larvae were incubated on NGM plates at 20°C until late L4 or early adult stage. Nematodes were exposed to H_2_O_2_ for 2 h, then the nematodes were transferred to the experimental plates [control group, 50 μM of CA, 2 mM of NAC (N-acetyl cysteine), or 20 mM of paraquat]. Nematodes were collected 96 h later and then processed with the ROS detection kit. After that, worms were observed and photographed (Hwang et al., 2014). For all worms, the images were analyzed using ImageJ software. At least 30 worms were used for each experiment. The experiment was repeated thrice. The *p* values were calculated using two-tailed Student’s t test.

### Determination of intracellular lipofuscin

Synchronized L1 stage N2 worms were raised at 20°C until they reached adulthood. Then, they were transferred to the NGM plates containing 50 μM of CA. The intestinal autofluorescence of lipofuscin was analyzed by using fluorescence microscopy (Leica DFC 7000T) on day 10 of adulthood (excitation wavelength, 360–370 nm; emission wavelength, 420–460 nm) (Höhn et al., 2010). The lipofuscin levels of each worm were quantified by determining the average pixel intensity of fluorescence in the intestine of each animal (except for the head and tail areas). At least 30 worms were used for each experiment. The experiment was repeated thrice. The *p* values were calculated using two-tailed Student’s t test.

### Fluorescence microscopic imaging

The treatment of worms with CA for fluorescence quantification was the same as the life span test. For strains Cl2166 *dvIs19* [(*pAF15*)gst-4p *::GFP::NLS*] *III*, CF1553 *muIs84* [(*pAD76*) sod-3p *::GFP +* rol-6(su1006)], SJ4100 *zcIs13*[hsp-6 *::GFP*], SJ4005 *zcIs4* [hsp-4 *::GFP*] *V*, and SJ4058 *zcIs9* [hsp-60 *::GFP +* lin-15(*+*)], the late L4 larvae or young adults were placed on plates with or without 50 μM CA at 20°C for 6 days. Late L4 larvae of LD1 *ldIs7* [skn-1b */c::GFP +* rol-6(su1006)] and TJ356 *zIs356* [daf-16p *::* daf-16a */b::GFP +* rol-6(su1006)] were placed on plates with 50 μM of CA or 20 mM of paraquat at 20°C for 4 h. Then, the worms were collected in a 1.5-ml centrifuge tube, washed with ddH_2_O thrice to remove OP50, then anesthetized with 2 mM of tetra-imidazole hydrochloride. The expression of GFP in these strains was observed by fluorescence microscope and photographed by using fluorescence microscope (Leica DFC 700T). Each group contained at least 30 worms. Finally, ImageJ was used to quantify the fluorescence intensity. The experiment was repeated independently at least thrice.

### Age-related neurodegenerative paralysis assay

Worms of CL4176 worms were incubated on the NGM plates containing 50 μM of CA from L1 larvae to the L4 stage at 15°C. Then, the worms were cultured at 25°C for 24 h (Wang et al., 2018). After that, paralyzed individuals were scored every 12 h. The L1 larvae of CL2006 were synchronized and incubated at 20°C on the NGM plates and incubated until late L4 or early adult stage. Then, the worms were transferred to the NGM plate with or without CA and counted and photographed every day. The worms that could not undergo full body wave propagation upon prodding or had halos were classified as paralyzed. The assay was repeated thrice with 60 worms per assay.

The L3 larvae of the transgenic strain BZ555 were transferred to a centrifuge tube containing 50 mM of 6-hydroxydopamine (6-OHDA) and 10 mM of ascorbic acid and incubated at 20°C for 1 h, and shaken gently every 10 min. Then, the worms were washed thrice with M9 buffer and incubated in petri dishes with and without CA for 72 h (Negga et al., 2012). Finally, the worms were washed with M9 and photographed. The experiments were repeated independently at least thrice. Each group of experiment included at least 30 individuals.

### Gene expression assay

Approximately 2,000 synchronized young adult worms were transferred to the NGM plates (9-cm diameter), with or without 50 μM of CA, and cultured at 20°C for 24 h. Total RNA was extracted by using RNAiso Plus (Takara) and converted to cDNA by using a High-Capacity cDNA Reverse Transcription Kit (Applied Biosystems). The qRT-PCR was performed in Power SYBR Green PCR Master Mix (Applied Biosystems) running in the QuantStudio 6 Flex system. The relative expression levels of the genes were evaluated using the 2^−ΔΔCT^ method and normalized to the expression of the gene *cdc-42*. The primers used here are listed in Supplementary Table S11. The *p* values were calculated using *t*-test.

## Results

### Complanatoside A extends life span of *Caenorhabditis elegans*

**FIGURE 1:**
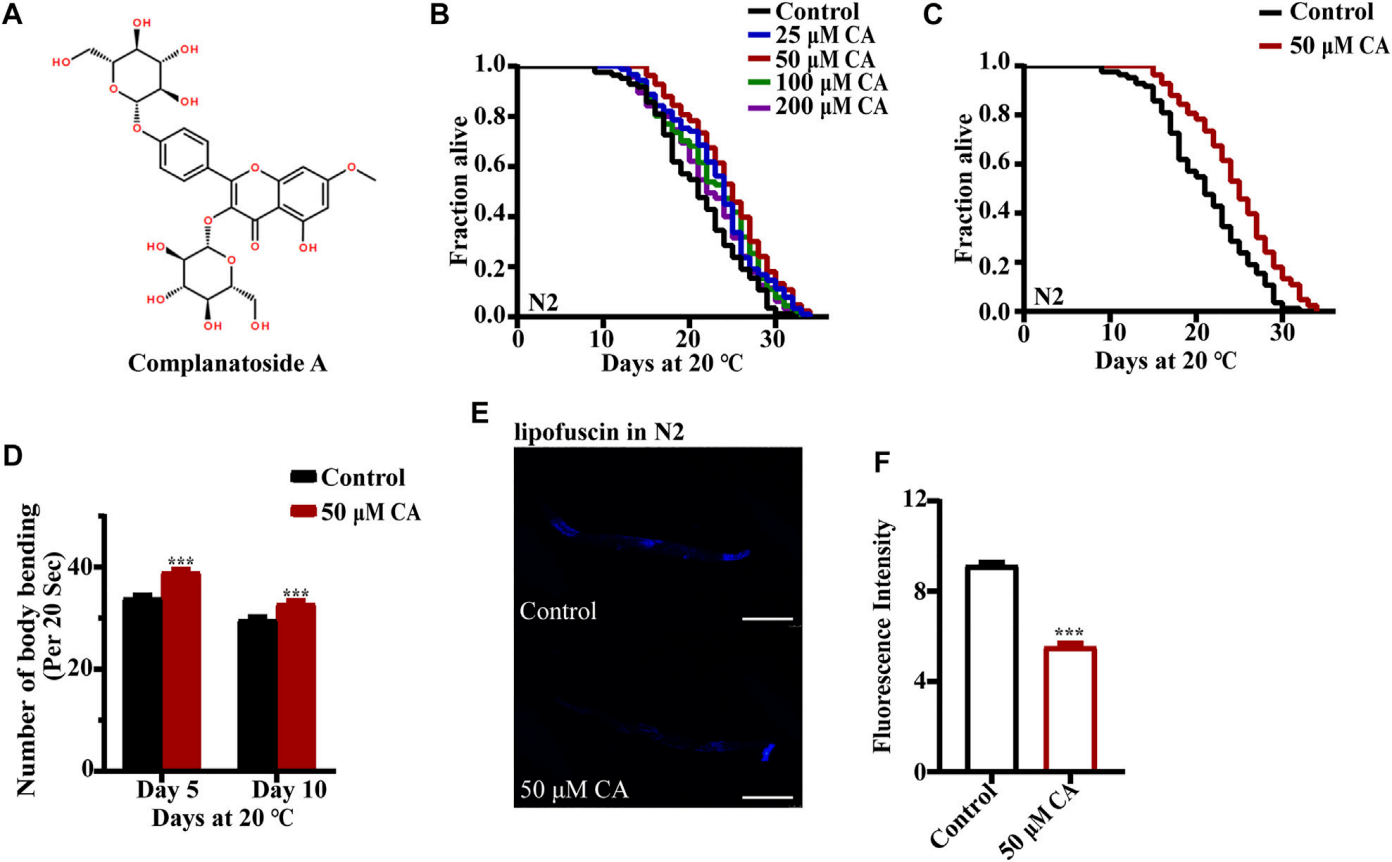
CA extends the life span of *C. elegans*. **(A)** Chemical structure of CA; **(B)** survival curves of wild-type N2 worms raised at 20°C on NGM plates containing either no CA or 25, 50, 100, and 200 μM of CA; **(C)** survival curves of wild-type N2 worms treated from hatching and raised at 20°C on NGM plates containing either no CA or 50 μM of CA (*p* < 0.001, log-rank test). Statistical details and repeats of these experiments are summarized in Supplementary Table S1. **(D)** Body bending behavior of nematode N2 treated with 50 μM of CA for 5 and 10 days. The figure is the mean value of three independent experiments, and the SEM is represented by an error line. *** represents *p* < 0.001, calculated by two-tailed *t*-test. Statistical details and repeats of these experiments are summarized in Supplementary Table S3. **(E)** The fluorescence picture of lipofuscin in N2 fed with or without 50 μM of CA on the 10th day of adulthood. **(F)** Analysis of lipofuscin in N2 nematodes. The relative fluorescence intensity was calculated by ImageJ. The figure is the mean value of three independent experiments; SEM is represented by error line. *** represents *p* < 0.001, calculated by two-tailed *t*-test. Statistical details and repeats of these experiments are summarized in Supplementary Table S2.

To determine whether CA can prolong the life span of *C. elegans*, we first treated wild-type nematode N2 with different concentrations of CA (Figure 1A). The results of multiple independent experiments showed that the longevity of *C. elegans* could be prolonged by different concentrations of CA, and 50 μM of which had the best effect, by extending the life span of the worms up to 16.87% (Figures 1B,C). Aging was associated with decreased exercise ability and accumulation of lipofuscin in the body (Stroikin et al., 2008; Gilbert et al., 2014). So, we investigated the effect of CA on the motor ability of *C. elegans* in the middle and old ages. Our results showed that CA significantly improved the locomotor capacity of *C. elegans* in middle and old ages (Figure 1D). CA also significantly reduced the content of lipofuscin in the worms on the 10th day of adulthood (Figures 1E,F).

### Complanatoside A delays amyloid-β–induced paralysis and reduces α-synuclein aggregation in *Caenorhabditis elegans*

**FIGURE 2:**
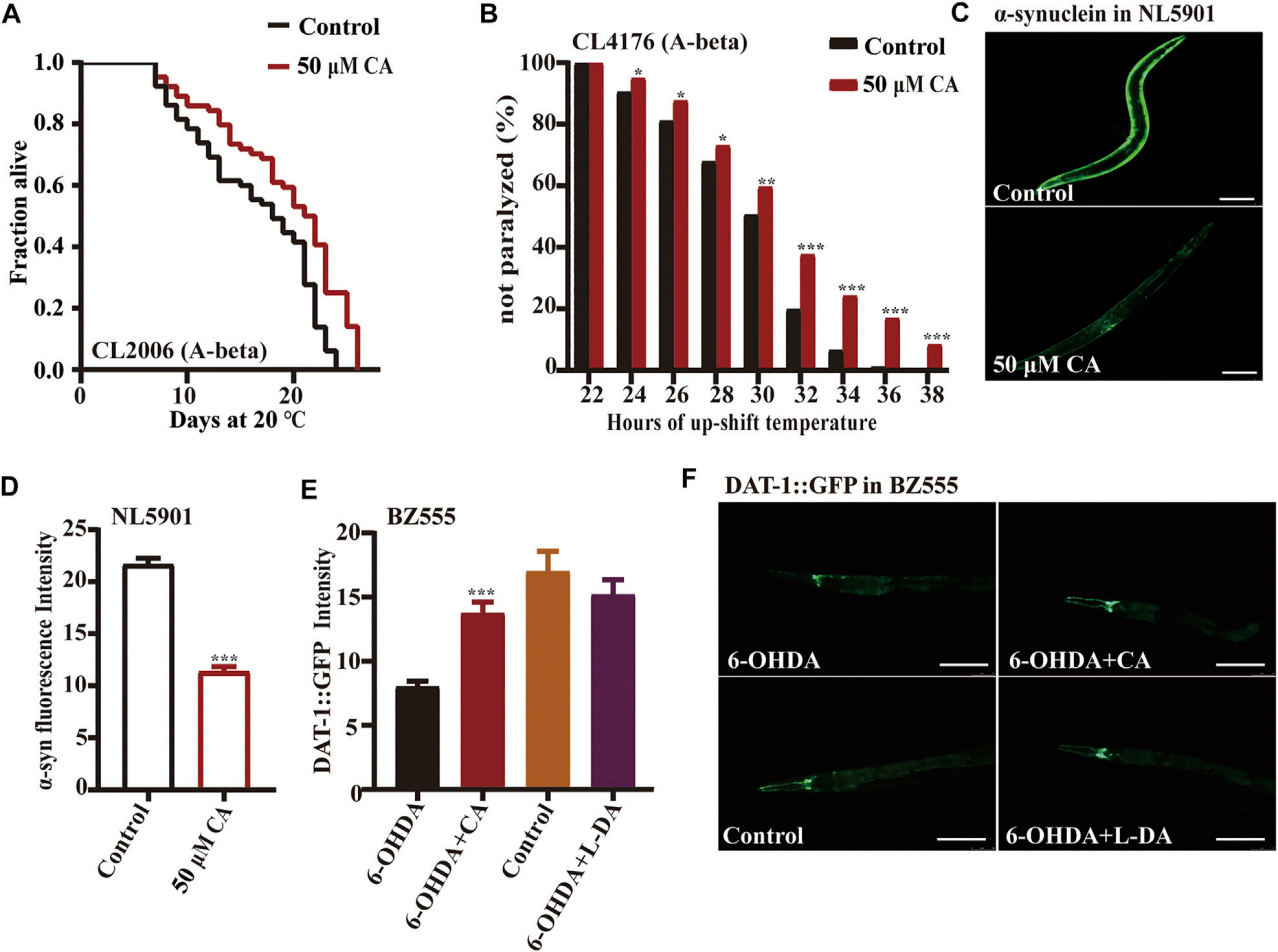
CA could delay the progression of neurodegenerative diseases in models of *C. elegans.* The effect of CA on the paralysis rate of nematodes CL2006 (A-beta) **(A)** and CL4176 (A-beta) **(B)** strains; **(C)** the fluorescence picture of YFP-labeled α-synuclein in *C. elegans* NL5901 fed with or without 50 μM of CA on the sixth day of adulthood. Analysis of the accumulation of α-synuclein in PD model nematodes NL5901 **(D)** and accumulation of dopamine transporter in BZ555 **(E)**. **(F)** The fluorescence picture of dopamine transporter conjugated with GFP in worms BZ555 fed with or without 50 μM of CA on the sixth day of adulthood, the positive control was used for levodopa. Statistical details and repeats of these experiments are summarized in Supplementary Table S8.

Alzheimer’s disease (AD) is an age-related neurodegenerative disorder characterized by chronic cognitive impairment and memory loss. There is limited understanding of the molecular pathobiology of AD, and no treatment is available (Mattson, 2004; Kundu et al., 2020). AD has two main characteristics, the extracellular amyloid-beta plaques and the intracellular neurofibrillary tangles those consist of highly phosphorylated tau protein (Kundu et al., 2020). The transgenic *C. elegans* CL2006 expressing human Aβ_1-42_ peptide constitutively in the muscle paralyze progressively beginning at adulthood (Diomede et al., 2013). While the expression of Aβ in strain CL4176 could be induced under the ambient temperature of 23°C in muscle cells (Wang et al., 2018), we found that the life span of transgenic nematode CL2006 treated with CA was longer than that of the control, and the paralysis rate of nematode CL4176 treated with CA was also significantly reduced under the elevated temperature (Figure 2A and Figure 2B).

The characteristic pathological feature of Parkinson’s disease (PD) is the Lewy body in the brain of patients. The aggregated α-synuclein is a major component of the Lewy body, and its continuous accumulation accelerates the developmental process of PD (Raza et al., 2019). Strain NL5901 expresses YFP-labeled α-synuclein in body wall muscles. The fluorescence intensity of the nematode body wall was considered a measure of α-synuclein aggregation (Govindan et al., 2018). Our results show that CA could significantly reduce the accumulation of α-synuclein in strain NL5901 (Figures 2C,D). The neurotoxin 6-hydroxy dopamine (6-OHDA) can cause the injury of dopaminergic neurons labeled by dopamine transporter conjugated with GFP in worms BZ555 *egIs1* [dat-1p *::GFP*] (McVey et al., 2016). Our results show that CA can restore nerve injury caused by 6-OHDA, which is similar to the improvement effect of the clinical medicine L-DA (levodopa) (Figures 2E,F).

### Complanatoside A improves oxidative and heat stress resistance of *Caenorhabditis elegans* by activating SKN-1 and HSF-1

**FIGURE 3:**
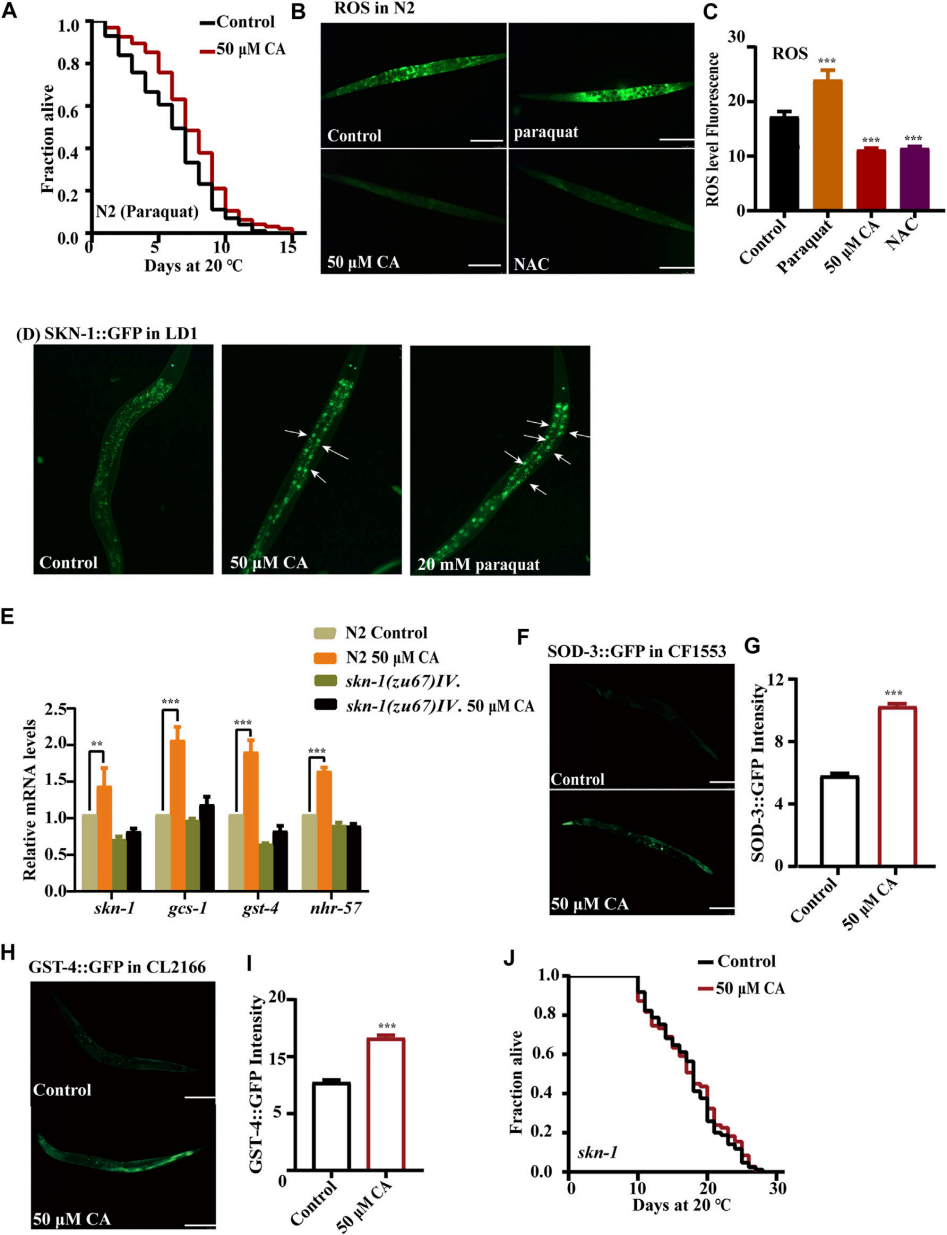
CA improves the oxidative stress resistance of *C. elegans* by activating SKN-1. **(A)** The survival percentage of wild-type worms cultured with 20 mM of paraquat and treated with 50 μM of CA (*p* < 0.001, log-rank test). Statistical details and repeats of these experiments are summarized in Supplementary Table S6. **(B)** ROS content detection of wild type N2 treated with 50 μM of CA. Paraquat (20 mM) and NAC (2 mM) are used as the negative and positive controls, respectively. Statistical details of these experiments are summarized in Supplementary Table S7. **(C)** Analysis of ROS in N2. **(D)** The fluorescence picture of SKN-1::GFP subcellular translocation in worms LD1 fed with or without 50 μM of CA on the L4 larvae or young adults. **(E)** Relative expression of downstream *skn-1* genes in L4 wild-type worms (N2) treated with CA for 24 h. Statistical details and repeats of these experiments are summarized in Supplementary Table S10. **(F)** The fluorescence picture of SOD-3::GFP in worms CF1553 fed with or without 50 μM of CA on the sixth day of adulthood. The quantification of fluorescence intensity of SOD-3::GFP **(G)** and GST-4::GFP **(H)**. **(I)** The fluorescence picture of GST-4::GFP in CL2166. **(J)** Survival curves of *skn-1*(*zu67*) raised at 20°C on the NGM plates containing CA in life span assays (*p* > 0.05). Statistical details of these experiments are summarized in Supplementary Table S4. CA significantly increased the expression of GST-4 and SOD-3; fluorescence intensity was calculated by Image J. The bar chart shows the mean value of three independently repeated experiments, and the error line represents SEM. *** represents *p* < 0.001, calculated by two-tailed *t*-test. Statistical details of these experiments are summarized in Supplementary Table S5.

Increased life span is usually accompanied by higher resistance to adverse environmental stressors. So, we investigated if CA could improve the resistance of *C. elegans* to oxidative and heat stresses. We found that CA could increase the survival rate of N2 worms treated with 20 mM of the oxidant paraquat (Figure 3A). While aging, the ability of the antioxidant defense system *in vivo* decreases and excessive accumulation of free radicals can cause tissue damage and organ degeneration (Shin et al., 2018)). Therefore, we detected the changes of ROS content in worms treated with CA. CA could significantly reduce the ROS level in N2 nematodes on day 10 of adulthood (Figures 3B,C). The transcription factor SKN-1 has a pivotal role in oxidative stress response, cellular homeostasis, and organismal life span (Dehghan et al., 2017). SKN-1 maintains the state of redox balance by regulating the expression of a variety of downstream antioxidant enzymes and phase II detoxifying enzymes, such as glutathione S-transferases (GSTs) (Settivari et al., 2013). Activated SKN-1 translocated into the nucleus, inducing the transcription of genes involved in oxidative stress response, which included genes encoding glutamate–cysteine ligase and glutathione S-transferases (Park et al., 2009). Our results have shown that CA treatment upregulated the accumulation of SKN-1::GFP in the nucleus (Figure 3D) and increased the mRNA levels of *skn-1* and its targeted genes such as *sod-3*, *gst-4*, *gcs-1*, and *nhr-57* (Figure 3E). CA also increased the protein levels of SOD-3 (superoxide dismutase 3) (Figures 3F,G) and GST-4 (glutathione S-transferase 4) (Figures 3H,I). Therefore, we further determined whether CA requires SKN-1 to prolong the longevity of *C. elegans.* We found that CA could not increase the longevity of the loss-of-function mutant EU1 *skn-1*(*zu67*) *IV* (Figure 3J).

**FIGURE 4:**
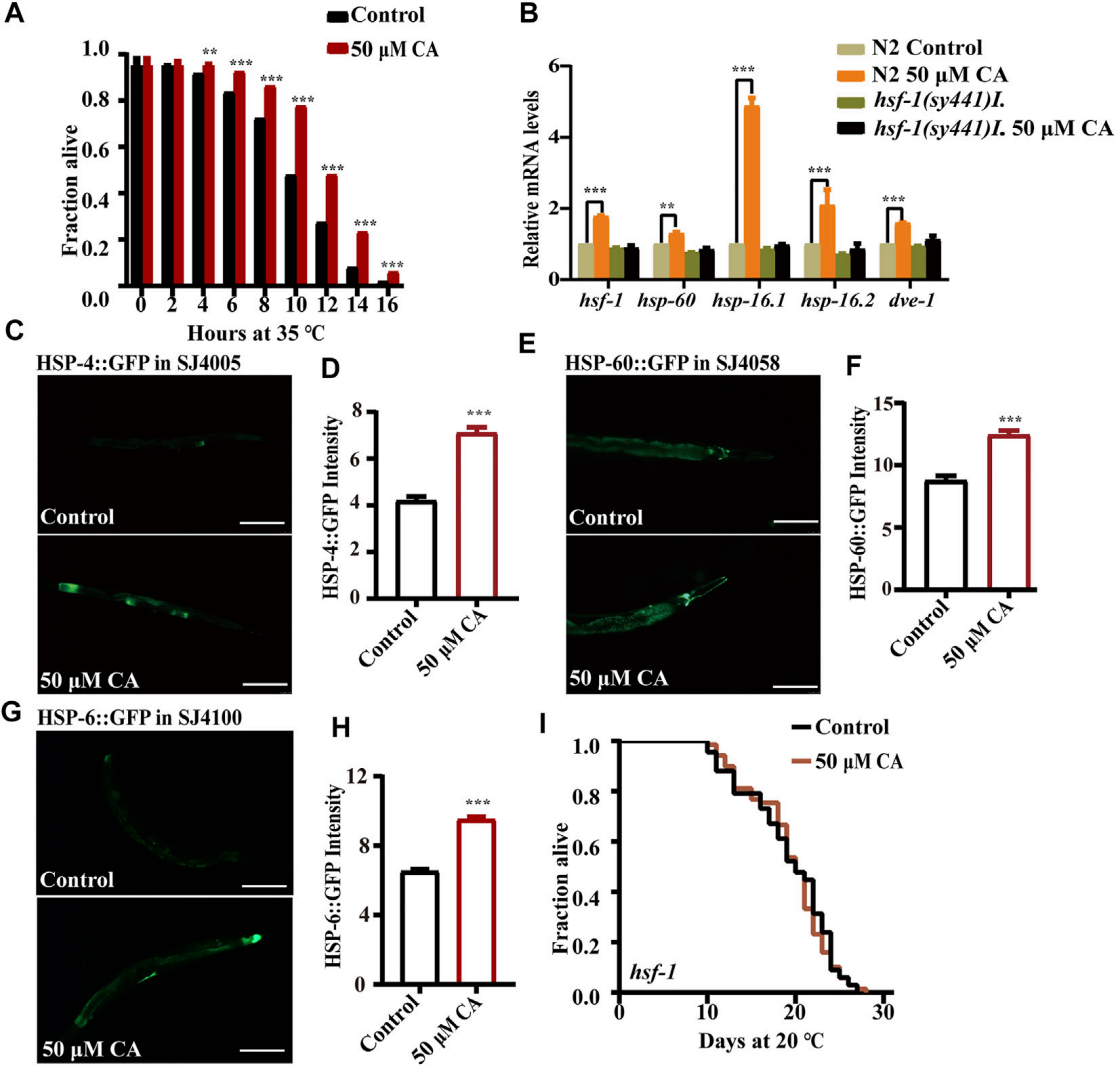
CA improves the heat stress resistance of *C. elegans* by activating HSF-1. **(A)** The survival percentage of wild-type worms treated with 50 μM of CA at 35°C (*p* < 0.001, log-rank test). Statistical details and repeats of these experiments are summarized in Supplementary Table S6. **(B)** Relative expression of downstream *hsf-1* genes in L4 wild-type worms (N2) treated with CA for 24 h. Statistical details and repeats of these experiments are summarized in Supplementary Table S10. The fluorescence picture of HSP-4::GFP **(C)**, HSP-60::GFP **(E)**, and HSP-6::GFP **(G)**. The quantification of fluorescence intensity of heat shock protein HSP-4 **(D)**, HSP-60 **(F)**, and HSP-6 **(H)**. **(I)** Survival curves of *hsf-1*(*sy441*)*I* raised at 20°C on NGM plates containing 50 μM of CA in life span assays (*p* > 0.05). Life span was analyzed using Kaplan–Meier analysis, and *p* values were calculated using log-rank test. Statistical details and repeats of these experiments are summarized in Supplementary Table S4. CA significantly increased the expressions of HSP-4, HSP-60, and HSP-6. Fluorescence intensity was calculated by ImageJ. The bar chart shows the mean value of three independently repeated experiments, and the error line represents SEM. *** represents *p* < 0.001. Statistical comparison analysis was calculated by two-tailed *t*-test. Statistical details and repeats of these experiments are summarized in Supplementary Table S5.

Heat stress could cause protein damage and aggregation and the formation of toxic protein oligomers, leading to perturbation of cellular function. We found that CA could significantly extend the survival of worms under the ambient temperature of 35°C (Figure 4A). The heat shock transcription factor HSF-1 is critical for heat stress response by regulating the expression of the chaperone heat shock proteins to maintain protein homeostasis (Dhondt et al., 2016). We found that CA significantly increased the transcription levels of the gene *hsf-1* and its regulated targeted genes encoding heat shock proteins (HSPs), such as *hsp-6*, *hsp-60*, *hsp-16.1*, *hsp-12.6* and *dve-1* (Figure 4B). In addition, CA also increased the expression of proteins HSP-4 (Figures 4C,D), HSP-60 (Figures 4E,F) ,and HSP-6 (Figures 4G,H) conjugated with GFP according to their fluorescent intensity. Moreover, we found that CA could not extend the life span of the mutant of *hsf-1* (Figure 4I).

### Complanatoside A requires transcription factor FOXO/DAF-16 to extend the longevity of *Caenorhabditis elegans*

**FIGURE 5:**
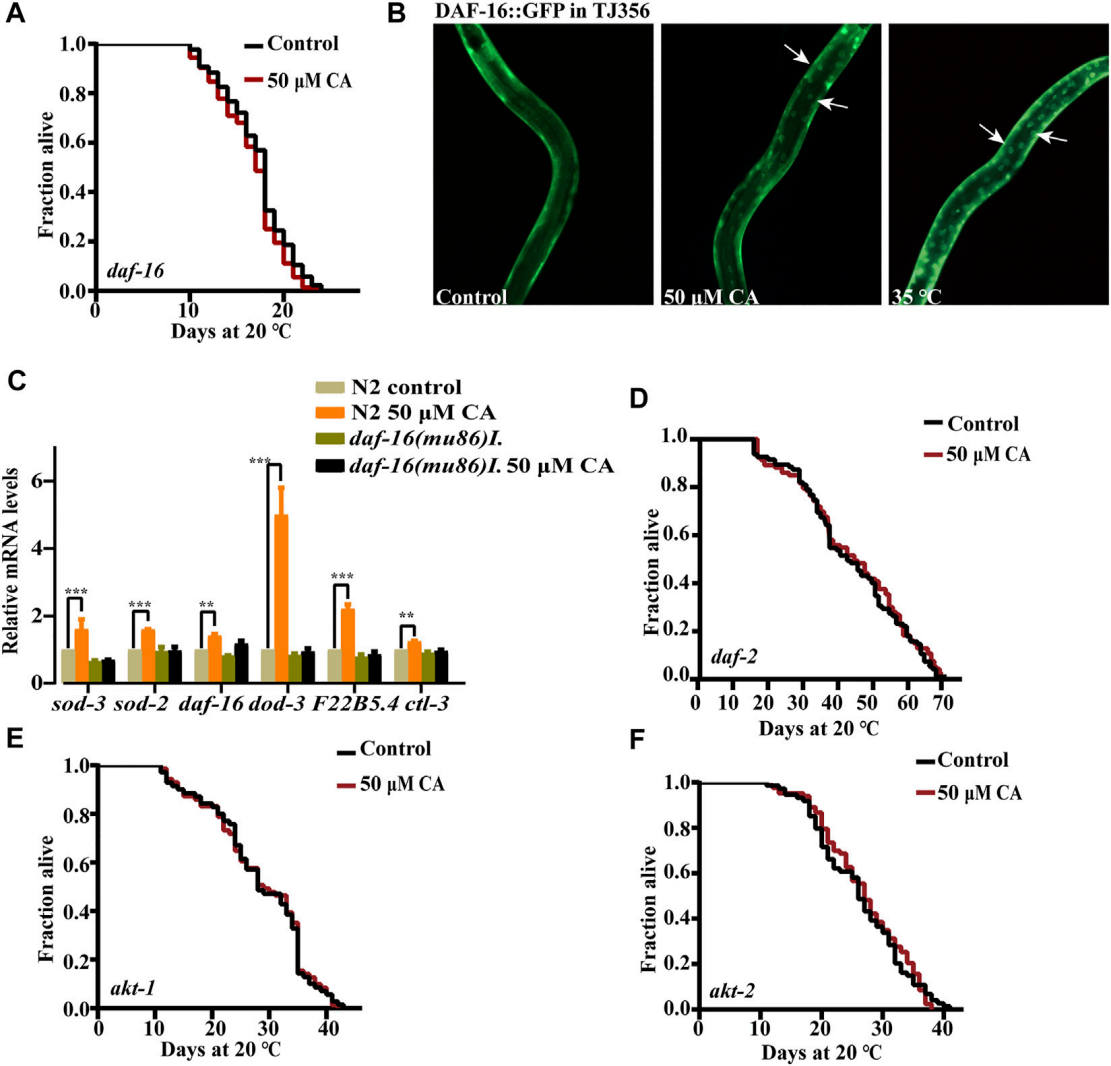
CA requires the transcription factor FOXO/DAF-16 to extend the longevity of *C. elegans*. **(A)** The survival curves of *daf-16*(*mu86*)*I* treated with CA in life span assays (*p* > 0.05). **(B)** CA induced DAF-16 nuclear localization. Worms of transgenic strain *daf-16*(*zls356*)*IV* were treated with CA at 20°C for 4 h. **(C)** Relative expression of downstream *daf-16* genes in L4 wild-type worms (N2) treated with CA for 24 h. Statistical details and repeats of these experiments are summarized in Supplementary Table S10. The survival curves of *daf-2*(*e1370*)*III* **(D)**, *akt-1*(*ok525*)*V* **(E)**, and *akt-2*(*ok393*)*X* **(F)** treated with CA in life span assays (*p* > 0.05), Statistical details and repeats of these experiments are summarized in Supplementary Table S4.

The forkhead box O (FOXO) transcription factor DAF-16 is the primary target of insulin/IGF-1 signaling and plays an important role in mediating stress resistance, development, reproduction, metabolism, and longevity (Zullo et al., 2019). So, we examined if CA could extend the life span of worms with the loss-of-function mutation in *daf-16*. We found that CA could not extend the life span of the *daf-16* null mutant *daf-16*(*mu86*) (Figure 5A), suggesting that the prolonged longevity of *C. elegans* induced by CA was dependent on *daf-16*. The inactive state DAF-16 is sequestered in the cytosol (McHugh et al., 2020). We investigated whether CA could stimulate the translocation of DAF-16 from the cytoplasm into the nucleus. The results showed that CA significantly increased the accumulation of DAF-16 in the nuclei of worms TJ356 (DAF-16::GFP) (Figure 5B). We found that CA also increased the mRNA levels of DAF-16–regulated genes such as *sod-3*, *sod-2*, *dod-3*, *ctl-3* and *F22B5.4* (Figure 5C) and the protein level of SOD-3 (Figures 3F,G), while the expression of *sod-3* was suppressed in *daf-16*(*mu86*). We then wonder if the components in the insulin/IGF-1 pathway upstream of DAF-16 could affect the effect of CA on longevity. We found that CA could not extend the life span of the deficient mutants of *daf-2*, *akt-1*, and *akt-2* (Figures 5D–F).

### Effect of Complanatoside A on energy processing pathways

**FIGURE 6:**
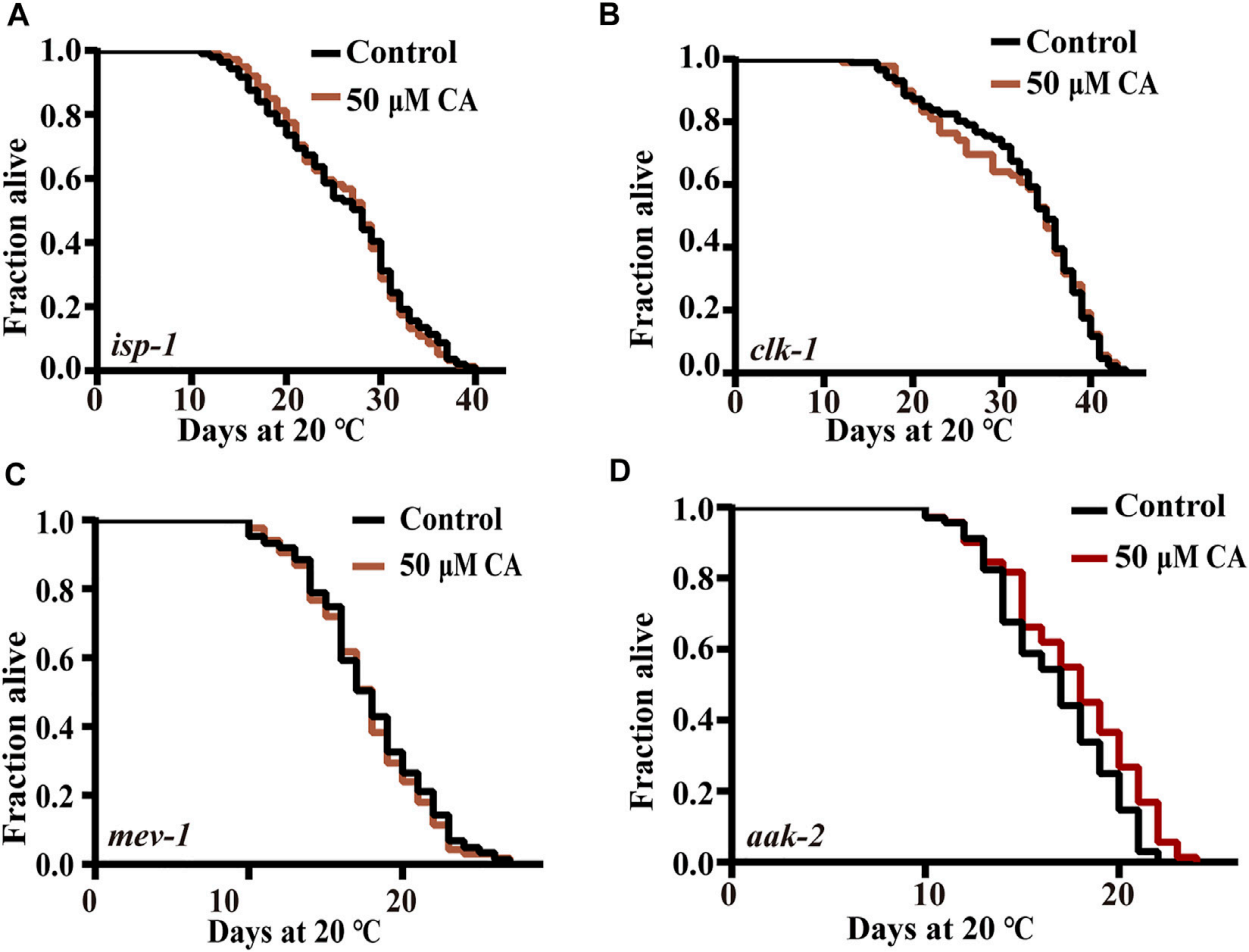
The effect of CA on energy processing pathways. The survival curves of *isp-1* (*qm150*) **(A)**, *clk-1*(*e2519*)*III* **(B)**, *mev-1*(*kn1*)*III* **(C)**, and *aak-2*(*ok524*)*X* **(D)** treated with CA in life span assays (*p* > 0.05). Life span was analyzed using Kaplan–Meier analysis, and *p* values were calculated using log-rank test. Statistical details and repeats of these experiments are summarized in Supplementary Table S4.

Alteration of the expression of certain subunits in the mitochondrial complex has been shown to significantly extend the life span of *C. elegans* (Zullo et al., 2019). These findings lead us to question whether mitochondrial function plays an important role in the longevity extension induced by CA. Our results showed that CA could not further extend the life span of the long-lived mutants of *isp-1* (encoding mitochondrial respiratory chain complex III), *clk-1* (homologous gene in human encoding coenzyme Q7, hydroxylase) (Khan et al., 2013), and the short-lived function-deficient mutant of *mev-1* (encoding the large cytochrome b subunit, Cyt-1/ceSDHC) (Feng et al., 2015) (Figure 6A, Figures 6B,C). AAK-2 is the catalytic subunit of AMPK, which senses the ratio of ATP/AMP and is also necessary for the longevity of *isp-1* and *clk-1* (Yang and Hekimi, 2010; Stroustrup et al., 2016; Kosztelnik et al., 2019). Our results showed that CA slightly extends the life span of *aak-2* mutant by about 6.31% (Figure 6D).

### Effect of Complanatoside A on reproductive signaling pathway

**FIGURE 7:**
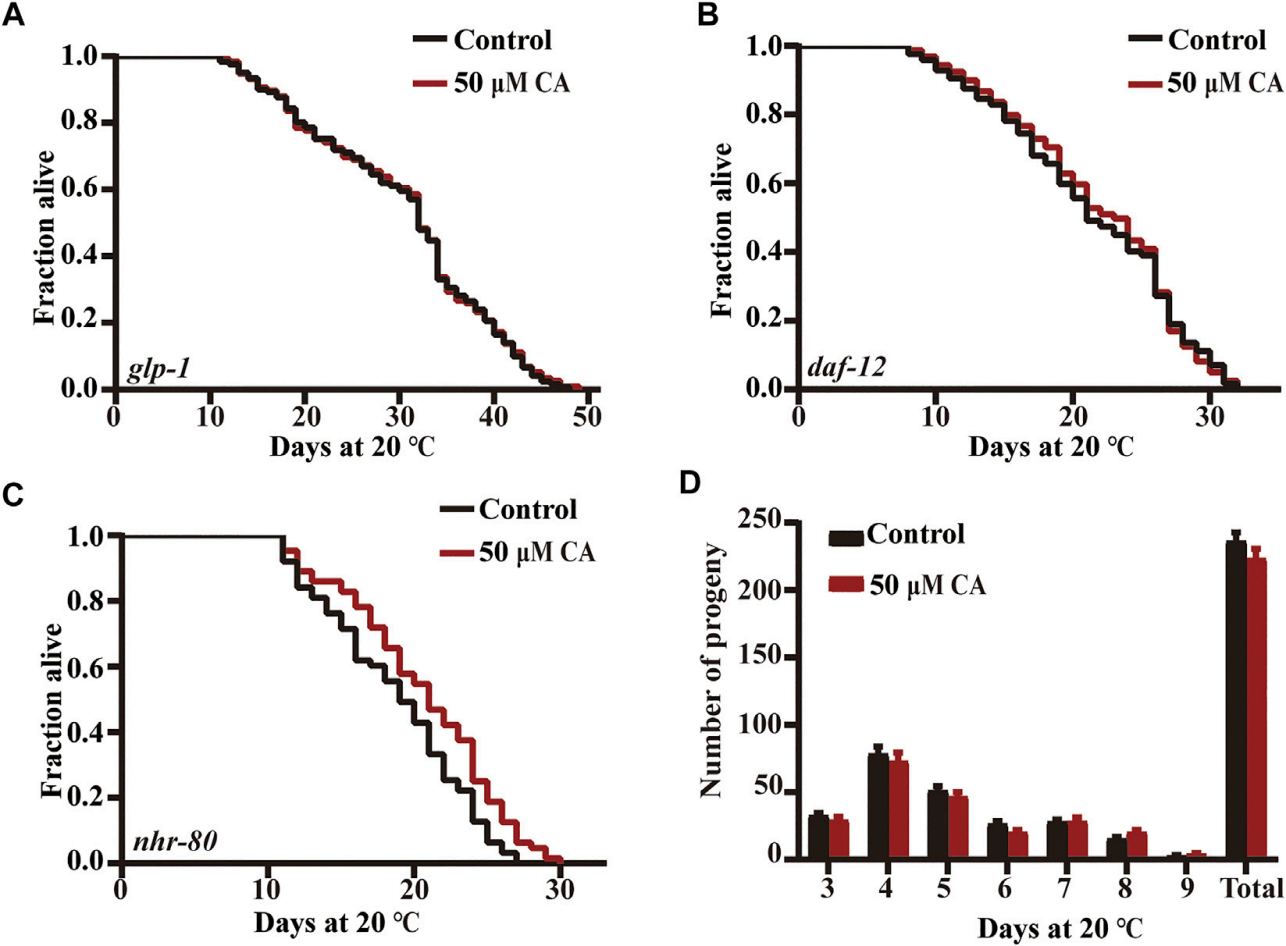
The effect of CA on the reproductive signaling pathway. The survival curves of *glp-1*(*e2141*)*III* **(A)**, *daf-12* (*rh274*) **(B)**, and *nhr-80*(*tm1011*)*III* **(C)** treated with CA in life span assays (*p* > 0.05). Life span was analyzed using Kaplan–Meier analysis, and *p* values were calculated using log-rank test. Statistical details and repeats of these experiments are summarized in Supplementary Table S4. **(D)** The effect of CA on the spawning rate of wild type N2. Statistical details and repeats of these experiments are summarized in Supplementary Table S9.

The removal of germ line stem cells can extend life span by about 60% *via* activating *daf-16* (Sinha and Rae, 2014). Germ stem cells promote fat accumulation and accelerate aging, while gonadal somatic cells produce a signal that delays aging and promotes lipolysis (Hansen et al., 2013; Farias-Pereira et al., 2020). The mutant CF1903 *glp-1*(*e2141*) *III* generates less germline stem cells under an ambient temperature (25°C) and is long lived. We found that CA could not prolong the life span of this mutant (Figure 7A).

Steroid hormone receptor DAF-12 regulates the diapause, development, and life span in *C. elegans* (Kaplan et al., 2015). The nuclear hormone receptor NHR-80 (HNF4 homolog) is necessary for the extended longevity in germ-free animals, and the overexpression of NHR-80/HNF4 can prolong the life of germ-free animals (Tillman et al., 2019). We found that CA could not extend *daf-12* defective mutant but could extend the life span of *nhr-80* deficient mutant (Figures 7B,C). We also showed that CA had no effect on the oviposition rate of nematodes (Figure 7D).

## Discussion

Here, we showed that CA could enhance the ability of *C. elegans* against stresses of oxidant paraquat and ambient high temperature. CA could also delay the progression of neurodegenerative diseases in models of *C. elegans* and extend the life span of *C. elegans*. Further investigation revealed that CA could increase the activity of antioxidation and detoxification regulated by SKN-1 and the expression of the chaperone heat shock proteins regulated by HSF-1 to maintain protein homeostasis. CA could also activate the transcription factor DAF-16. The above results suggest that CA could activate multiple cellular protective pathways and is an antiaging agent worth further research for the development of pharmaceutical application.

Our results showed that CA could activate the transcription factor DAF-16, which is also required for CA to extend the life span of *C. elegans*. The genes *daf-2*, *akt-1*, and *akt-2* encode the representative molecules in the insulin/IGF-1 pathway to inhibit the activities of DAF-16. CA could not further extend the life span of the deficient mutants of *daf-2*, *akt-1*, and *akt-2*. The transcription factors SKN-1 and HSF-1 are critical for the regulation of stress responses such as immune response, oxidative response, heat shock response, and protein homeostasis (Zečić and Braeckman, 2020). Our results showed that CA could activate SKN-1 and HSF-1. SKN-1 and HSF-1 are also required for CA to extend the life span of *C. elegans*. In the long-lived *daf-2* mutant, the SKN-1 and HSF-1 are accumulated in the nucleus and activated (Hsu et al., 2003; Tullet et al., 2008). In *daf-2* mutant, HSF-1 is responsible for increased proteostasis, while SKN-1 is responsible for increased oxidative stress resistance (Lapierre and Hansen, 2012). The above results indicate that CA might act through the insulin/IGF-1 pathway to increase the stress resistance and improve the life span of *C. elegans*.

We found that CA could extend the life span of *nhr-80*–deficient mutant and moderately extend the life span of the mutant *aak-2*, suggesting that the metabolism pathways might not play a role in the effect of CA on life span. CA could also not extend the life span of the mutants of *isp-1*, *clk-1*, *glp-1*, and *daf-12*. These results suggest that either the effect of CA acts on the same pathway of these genes or the effect of CA is not strong enough to further extend the already extended life span of these mutants. Further investigations are needed to fully understand the effect of CA on these pathways.

## Conclusion

Our results revealed that CA could enhance the stress resistances of *C. elegans*, delay neurodegenerative diseases, and have antioxidant and antiaging activities by activating multiple cellular protection pathways. Since CA has been widely used in humans for a long time, our results suggest that CA might be a promising antiaging candidate that is worth further research on its pharmacological mechanism and pharmaceutical applications.

## Data Availability

The original contributions presented in the study are included in the article/Supplementary Material; further inquiries can be directed to the corresponding authors.
