# Supplementary material for: Flavonol glycoside complanatoside A requires FOXO/DAF-16, NRF2/SKN-1, and HSF-1 to improve stress resistances and extend the life span of Caenorhabditis elegans
Source: Front Pharmacol. 2022 Aug 22;13:931886. doi: 10.3389/fphar.2022.931886 (PMC9441740; doi:10.3389/fphar.2022.931886)
Supplement: Supplementary file 1 [file DataSheet1.docx]

Flavonol Glycoside Complanatoside A Requires FOXO/DAF-16, NRF2/SKN-1, and HSF-1 to Improve Stress Resistances and Extend the Lifespan of *C. elegans*

Lin Tan^1,2^, Zhuo-Ya Zheng^1^, Lv Huang^1^, Zhong Jin^3^, Su-Lian Li^4^, Gui-Sheng Wu^1, 4, 5*^, Huai-Rong Luo^1, 5, 6*^

^1^ Key Laboratory for Aging and Regenerative Medicine, Department of Pharmacology School of Pharmacy, Southwest Medical University, Luzhou, Sichuan 646000, China

^2^ Department of Pharmacy, Guang'an People's Hospital, Guang'an, Sichuan 638550, China

^3^ Luzhou City Hospital of Traditional Chinese Medicine, Luzhou, Sichuan 646000, China

^4^ Affiliated Traditional Chinese Medicine Hospital of Southwest Medical University, Luzhou, Sichuan, 646000, China

^5^ Central Nervous System Drug Key Laboratory of Sichuan Province, Luzhou, Sichuan 646000, China

^6^ Key Laboratory of Medical Electrophysiology, Ministry of Education & Medical Electrophysiological Key Laboratory of Sichuan, Institute of Cardiovascular Research, Southwest Medical University, Luzhou, Sichuan 646000, China

* Corresponding author: Dr. Huai-Rong Luo, Dr. Gui-Sheng Wu

Key Laboratory for Aging and Regenerative Medicine, Department of Pharmacology

School of Pharmacy, Southwest Medical University

1 Xianglin Road, Luzhou, Sichuan 646000, China

Phone: +86 830-3160842; Fax: +86 830-3160842

E-mail address: [lhr@swmu.edu.cn](mailto:lhr@swmu.edu.cn), [wgs@swmu.edu.cn](mailto:wgs@swmu.edu.cn)

ORCID: <https://orcid.org/0000-0001-8912-6694>, <https://orcid.org/0000-0003-1913-9566>

**Supplementary materials**

**Contents:**

**Table S1. Lifespan of wild-type nematode (N2) treated with CA in different concentrations.**

**Table S2. Effect of CA on lipofuscin in wild-type nematode (N2).**

**Table S3. Effect of CA on body movement in wild-type nematode (N2).**

**Table S4. Effect of CA on lifespan of mutant nematode.**

**Table S5. Effect of CA on expression of SOD-3, GST-4, HSP-4, HSP-6, HSP-60 in mutant nematode*.***

**Table S6. Effect of CA on resistance to high temperature and oxidation in wild-type nematode (N2).**

**Table S7. Effect of CA on ROS level of N2 nematode.**

**Table S8. Effects of CA on age-related diseases.**

**Table S9. Effect of CA on oviposition rate of N2 worm.**

**Table S10. Effect of CA on gene expression at mRNA level in nematode.**

**Table S11. Primers used for the analysis of gene expression in nematode.**

**Table S1. Lifespan of wild-type nematode (N2) treated with CA in different concentrations.**

| Figure 1B |  | Control (0μM) | 25μM  CA | 50μM  CA | 100μM  CA | 200μM  CA |
| --- | --- | --- | --- | --- | --- | --- |
| Concentration |  |  |  |  |  |  |
| strain |  | **N2(WT)** | **N2(WT)** | **N2(WT)** | **N2(WT)** | **N2(WT)** |
|  |  |  |  |  |  |  |
| Treatments |  | 20℃/OP50  (dead) | 20℃/OP50  (dead) | 20℃/OP50  (dead) | 20℃/OP50  (dead) | 20℃/OP50  (dead) |
| Mean±SEM | EXP.1 | 21.131±0.580 | 23.360±0.567 | 24.651±0.555 | 23.000±0.584 | 22.347±0.551 |
|  | EXP.2 | 20.163±0.468 | 22.753±0.575 | 23.653±0.561 | 22.070±0.610 | 21.380±0.479 |
|  | EXP.3 | 20.487±0.494 | 22.356±0.578 | 23.922±0.625 | 22.471±0.588 | 21.346±0.628 |
|  | EXP.4 | 22.140±0.559 | 24.602±0.581 | 25.849±0.538 | 23.989±0.527 | 23.659±0.654 |
|  |  |  |  |  |  |  |
| *P* value  VS control | EXP.1 |  | <0.0001 | <0.0001 | <0.001 | <0.05 |
|  | EXP.2 |  | <0.0001 | <0.0001 | <0.001 | <0.05 |
|  | EXP.3 |  | <0.0001 | <0.0001 | <0.001 | <0.05 |
|  | EXP.4 |  | <0.0001 | <0.0001 | <0.001 |  |
|  |  |  |  |  |  |  |
| N | EXP.1 | 84 | 89 | 83 | 91 | 95 |
|  | EXP.2 | 80 | 73 | 75 | 71 | 71 |
|  | EXP.3 | 78 | 73 | 77 | 70 | 81 |
|  | EXP.4 | 86 | 83 | 93 | 90 | 85 |
|  |  |  |  |  |  |  |
| change in mean lifespan | EXP.1 |  | 10.55% | 16.66% | 8.85% | 5.76% |
|  | EXP.2 |  | 12.85% | 17.31% | 9.46% | 6.04% |
|  | EXP.3 |  | 9.12% | 16.77% | 9.68% | 4.19% |
|  | EXP.4 |  | 11.12% | 16.75% | 8.35% | 6.86% |
|  |  |  |  |  |  |  |

*p*-value was analyzed by log-rank (Mantel-Cox) test.

N: number of dead worms.

*P* <0.05 indicated that the experiment was statistically significant, while *P* >0.05 indicated that the experiment was not statistically significant.

**Table S2. Effect of CA on lipofuscin in wild-type nematode (N2).**

| Figure | Strain | Treatments | Mean Pigment  ±SEM | *P* value  VS  control | N |
| --- | --- | --- | --- | --- | --- |
| 1E, 1F | **N2(WT)** | OP50(dead) |  |  |  |
|  | EXP.1 | 20℃/control | 9.157±0.127 | <0.0001 | 30 |
|  | EXP.1 | 20℃/50μM CA | 5.548±0.148 |  | 30 |
|  | EXP.2 | 20℃/control | 8.316±0.153 | <0.0001 | 30 |
|  | EXP.2 | 20℃/50μM CA | 4.419±0.151 |  | 30 |
|  | EXP.3 | 20℃/control | 9.399±0.150 | <0.0001 | 30 |
|  | EXP.3 | 20℃/50μM CA | 6.302±0.122 |  | 32 |
|  |  |  |  |  |  |

On the 10th day of the adult worm, the accumulation of lipofuscin in intestinal tissues of the treated and untreated nematodes was photographed and counted. Fluorescence intensity was analyzed by Image J, and *p* value was calculated by two-tailed t-test, where *P* <0.05 indicated that the experiment was statistically significant

**Table S3. Effect of CA on body movement in wild-type nematode (N2).**

| Figure | Strain | Treatment | Mean Pigment  ±SEM | P value  VS  control | N | Mean Pigment  ±SEM | P value  VS  control | N |
| --- | --- | --- | --- | --- | --- | --- | --- | --- |
| 1D | **N2(WT)** | OP50(dead) | 5 day of adult |  |  | | | |
|  | EXP.1 | 20℃/control | 33.967±0.492 | <0.001 | 30 | 29.733± 0.503 | <0.001 | 30 |
|  | EXP.1 | 20℃/50μM CA | 39.067±0.486 |  | 30 | 32.867± 0.573 |  |  |
|  | EXP.2 | 20℃/control | 32.067±0.542 | <0.0001 | 30 | 27.033±0.448 | <0.0001 | 30 |
|  | EXP.2 | 20℃/50μM CA | 37.333±0.586 |  | 30 | 30.233±0.666 |  |  |
|  | EXP.3 | 20℃/control | 34.167±0.597 | <0.0001 | 30 | 29.467±0.717 | <0.0001 | 30 |
|  | EXP.3 | 20℃/50μM CA | 39.167±0.576 |  | 30 | 33.133±0.621 |  |  |
|  |  |  |  |  |  |  |  |  |

The body movement count time was 20 seconds per nematode. N was the experimental sample size, and P value was calculated by two-tailed t-test. *P* <0.05 indicated that the experiment was statistically significant

**Table S4. Effect of CA on lifespan of mutant nematode.**

| Figure | Strain | Treatment | Mean lifespan  ±SEM  (days) | P value  VS  control | change in mean lifespan | N |
| --- | --- | --- | --- | --- | --- | --- |
| 5D | **CF1370**  ***daf-2 (e1370)Ⅲ.*** | OP50(dead) | Days |  |  |  |
|  |  |  |  |  |  |  |
|  | EXP.1 | 20℃/control | 44.835±1.676 | 0.7 | # | 79 |
|  | EXP.1 | 20℃/50μM CA | 45.688±1.698 |  |  | 77 |
|  | EXP.2 | 20℃/control | 44.224±1.689 | 0.286 | # | 81 |
|  | EXP.2 | 20℃/50μM CA | 45.224±1.982 |  |  | 85 |
|  | EXP.3 | 20℃/control | 43.453±1.530 | 0.616 | # | 95 |
|  | EXP.3 | 20℃/50μM CA | 44.075±1.611 |  |  | 93 |
|  |  |  |  |  |  |  |
| 5E | **RB759**  ***akt-1 (ok525)V.*** | OP50(dead) |  |  |  |  |
|  |  |  |  |  |  |  |
|  | EXP.1 | 20℃/control | 27.758±0.757 | 0.349 | # | 91 |
|  | EXP.1 | 20℃/50μM CA | 28.964±0.756 |  |  | 83 |
|  | EXP.2 | 20℃/control | 28.680±0.947 | 0.986 | # | 75 |
|  | EXP.2 | 20℃/50μM CA | 28.437±1.056 |  |  | 71 |
|  | EXP.3 | 20℃/control | 28.300±1.008 | 0.986 | # | 70 |
|  | EXP.3 | 20℃/50μM CA | 28.338±1.018 |  |  | 71 |
|  |  |  |  |  |  |  |
| 5F | **VC204**  ***akt-2 (ok393)X.*** | OP50(dead) |  |  |  |  |
|  |  |  |  |  |  |  |
|  | EXP.1 | 20℃/control | 25.463±0.763 | 0.942 | # | 82 |
|  | EXP.1 | 20℃/50μM CA | 25.973±0.822 |  |  | 73 |
|  | EXP.2 | 20℃/control | 26.311±0.841 | 0.876 | # | 74 |
|  | EXP.2 | 20℃/50μM CA | 27.084±0.764 |  |  | 83 |
|  | EXP.3 | 20℃/control | 25.176±0.603 | 0.135 | # | 74 |
|  | EXP.3 | 20℃/50μM CA | 26.194±0.631 |  |  | 93 |
|  |  |  |  |  |  |  |
| 5A | **CF1038**  ***daf-16 (mu86)Ⅰ.*** | OP50(dead) |  |  |  |  |
|  |  |  |  |  |  |  |
|  | EXP.1 | 20℃/control | 18.367±0.585 | 0.764 | # | 79 |
|  | EXP.1 | 20℃/50μM CA | 17.727±0.588 |  |  | 88 |
|  | EXP.2 | 20℃/control | 18.158±0.411 | 0.311 | # | 95 |
|  | EXP.2 | 20℃/50μM CA | 17.615±0.405 |  |  | 91 |
|  | EXP.3 | 20℃/control | 17.852±0.605 | 0.451 | # | 61 |
|  | EXP.3 | 20℃/50μM CA | 18.297±0.650 |  |  | 64 |
|  | EXP.4 | 20℃/control | 17.221±0.374 | 0.169 | # | 86 |
|  | EXP.4 | 20℃/50μM CA | 16.569±0.397 |  |  | 72 |
|  |  |  |  |  |  |  |
| 4I | **PS3551**  ***hsf-1 (sy441)Ⅰ.*** | OP50(dead) |  |  |  |  |
|  |  |  |  |  |  |  |
|  | EXP.1 | 20℃/control | 18.410±0.656 | 0.264 | # | 61 |
|  | EXP.1 | 20℃/50μM CA | 19.788±0.610 |  |  | 66 |
|  | EXP.2 | 20℃/control | 19.065±0.458 | 0.110 | # | 108 |
|  | EXP.2 | 20℃/50μM CA | 19.757±0.520 |  |  | 103 |
|  | EXP.3 | 20℃/control | 19.284±0.592 | 0.716 | # | 67 |
|  | EXP.3 | 20℃/50μM CA | 19.333±0.532 |  |  | 69 |
|  |  |  |  |  |  |  |
| 3J | **EU1**  ***skn-1 (zu67).*** | OP50(dead) |  |  |  |  |
|  |  |  |  |  |  |  |
|  | EXP.1 | 20℃/control | 16.694±0.558 | 0.97 | # | 72 |
|  | EXP.1 | 20℃/50μM CA | 16.895±0.533 |  |  | 67 |
|  | EXP.2 | 20℃/control | 17.565±0.533 | 0.653 | # | 85 |
|  | EXP.2 | 20℃/50μM CA | 17.718±0.632 |  |  | 71 |
|  | EXP.3 | 20℃/control | 17.649±0.583 | 0.878 | # | 94 |
|  | EXP.3 | 20℃/50μM CA | 17.830±0.511 |  |  | 94 |
|  | EXP.4 | 20℃/control | 16.778±0.702 | 0.575 | # | 63 |
|  | EXP.4 | 20℃/50μM CA | 16.803±0.582 |  |  | 71 |
|  |  |  |  |  |  |  |
| 6A | **MQ887**  ***isp-1 (qm150)IV.*** | OP50(dead) |  |  |  |  |
|  |  |  |  |  |  |  |
|  | EXP.1 | 20℃/control | 26.130±0.523 | 0.723 | # | 193 |
|  | EXP.1 | 20℃/50μM CA | 26.347±0.457 |  |  | 213 |
|  | EXP.2 | 20℃/control | 27.855±0.499 | 0.793 | # | 194 |
|  | EXP.2 | 20℃/50μM CA | 28.246±0.476 |  |  | 191 |
|  | EXP.3 | 20℃/control | 25.63±0.493 | 0.895 | # | 133 |
|  | EXP.3 | 20℃/50μM CA | 24.772±0.493 |  |  | 145 |
|  |  |  |  |  |  |  |
| 6B | **CB4876**  ***clk-1 (e2519)III.*** | OP50(dead) |  |  |  |  |
|  |  |  |  |  |  |  |
|  | EXP.1 | 20℃/control | 32.744±0.835 | 0.996 | # | 86 |
|  | EXP.1 | 20℃/50μM CA | 32.079±0.866 |  |  | 89 |
|  | EXP.2 | 20℃/control | 32.822±1.015 | 0.518 | # | 73 |
|  | EXP.2 | 20℃/50μM CA | 31.074±1.075 |  |  | 68 |
|  | EXP.3 | 20℃/control | 33.087±0.674 | 0.875 | # | 115 |
|  | EXP.3 | 20℃/50μM CA | 32.099±0.753 |  |  | 111 |
|  |  |  |  |  |  |  |
| 6C | **TK22**  ***mev-1 (kn1)III.*** | OP50(dead) |  |  |  |  |
|  |  |  |  |  |  |  |
|  | EXP.1 | 20℃/control | 18.506±0.402 | 0.239 | # | 77 |
|  | EXP.1 | 20℃/50μM CA | 17.736±0.402 |  |  | 72 |
|  | EXP.2 | 20℃/control | 17.857±0.327 | 0.543 | # | 147 |
|  | EXP.2 | 20℃/50μM CA | 17.629±0.293 |  |  | 167 |
|  | EXP.3 | 20℃/control | 17.284±0.373 | 0.297 | # | 102 |
|  | EXP.3 | 20℃/50μM CA | 17.949±0.335 |  |  | 117 |
|  |  |  |  |  |  |  |
| 6D | **RB754**  ***aak-2 (ok524)X.*** | OP50(dead) |  |  |  |  |
|  |  |  |  |  |  |  |
|  | EXP.1 | 20℃/control | 16.676±0.388 | 0.017 | 5.83% | 68 |
|  | EXP.1 | 20℃/50μM CA | 17.648±0.425 |  |  | 71 |
|  | EXP.2 | 20℃/control | 17.107±0.353 | 0.003 | 6.30% | 75 |
|  | EXP.2 | 20℃/50μM CA | 18.184±0.45 |  |  | 87 |
|  | EXP.3 | 20℃/control | 16.581±0.345 | 0.011 | 6.80% | 115 |
|  | EXP.3 | 20℃/50μM CA | 17.708±0.393 |  |  | 111 |
|  |  |  |  |  |  |  |
| 7A | **CF1903**  ***glp-1***  ***(e2141)III.*** | OP50(dead) |  |  |  |  |
|  |  |  |  |  |  |  |
|  | EXP.1 | 20℃/control | 30.397±0.878 | 0.818 | # | 121 |
|  | EXP.1 | 20℃/50μM CA | 30.448±0.904 |  |  | 116 |
|  | EXP.2 | 20℃/control | 29.046±1.004 | 0.951 | # | 109 |
|  | EXP.2 | 20℃/50μM CA | 29.303±1.020 |  |  | 99 |
|  | EXP.3 | 20℃/control | 30.946±1.069 | 0.788 | # | 74 |
|  | EXP.3 | 20℃/50μM CA | 30.475±1.123 |  |  | 80 |
|  |  |  |  |  |  |  |
| 7B | **AA89**  ***daf-12 (rh274).*** | OP50(dead) |  |  |  |  |
|  |  |  |  |  |  |  |
|  | EXP.1 | 20℃/control | 21.343±0.508 | 0.904 | # | 169 |
|  | EXP.1 | 20℃/50μM CA | 21.767±0.500 |  |  | 159 |
|  | EXP.2 | 20℃/control | 21.552±0.551 | 0.240 | # | 174 |
|  | EXP.2 | 20℃/50μM CA | 20.890±0.558 |  |  | 164 |
|  | EXP.3 | 20℃/control | 21.112±0.505 | 0.706 | # | 89 |
|  | EXP.3 | 20℃/50μM CA | 21.286±0.489 |  |  | 98 |

|  |  |  |  |  |  |  |
| --- | --- | --- | --- | --- | --- | --- |
| 7C | **BX165**  ***nhr-80 (tm1011)III.*** | OP50(dead) |  |  |  |  |
|  |  |  |  |  |  |  |
|  | EXP.1 | 20℃/control | 19.112±0.550 | 0.003 | 9.23 | 98 |
|  | EXP.1 | 20℃/50μM CA | 20.876±0.589 |  |  | 113 |
|  | EXP.2 | 20℃/control | 18.778±0.608 | 0.014 | 9.84 | 63 |
|  | EXP.2 | 20℃/50μM CA | 20.625±0.643 |  |  | 64 |
|  | EXP.3 | 20℃/control | 18.830±0.556 | 0.01 | 7.37 | 88 |
|  | EXP.3 | 20℃/50μM CA | 20.218±0.646 |  |  | 87 |
|  |  |  |  |  |  |  |

***p***-value was analyzed by log-rank (Mantel-Cox) test.

N: number of dead worms.

*P* <0.05 indicated that the experiment was statistically significant, while *P* >0.05 indicated that the experiment was not statistically significant.

**Table S5. Effect of CA on expression of SOD-3, GST-4, HSP-4, HSP-6, HSP-60 in mutant nematode*.***

| Figure | Strain | Treatment | Fluorescence  intensity  Mean±SD | P value  VS  Control | N |
| --- | --- | --- | --- | --- | --- |
| 3F, 3G | **CF1553**  ***pAD76* (SOD-3::GFP)** | OP50(dead) |  |  |  |
|  |  |  |  |  |  |
|  | EXP.1 | 20℃/control | 5.811±0.151 | <0.0001 | 30 |
|  | EXP.1 | 20℃/50μM CA | 10.253±0.155 |  | 30 |
|  | EXP.2 | 20℃/control | 5.277±0.141 | <0.0001 | 34 |
|  | EXP.2 | 20℃/50μM CA | 9.948±0.280 |  | 33 |
|  | EXP.3 | 20℃/control | 5.233±0.158 | <0.0001 | 36 |
|  | EXP.3 | 20℃/50μM CA | 8.526±0.152 |  | 34 |
|  |  |  |  |  |  |
| 3H, 3I | **CL2166**  ***dvIs19* (GST-4::GFP)** | OP50(dead) |  |  |  |
|  |  |  |  |  |  |
|  | EXP.1 | 20℃/control | 7.704±0.436 | <0.0001 | 42 |
|  | EXP.1 | 20℃/50μM CA | 12.153±0.620 |  | 34 |
|  | EXP.2 | 20℃/control | 8.123±0.327 | <0.0001 | 46 |
|  | EXP.2 | 20℃/50μM CA | 12.825±0.640 |  | 46 |
|  | EXP.3 | 20℃/control | 7.743±0.201 | <0.0001 | 41 |
|  | EXP.3 | 20℃/50μM CA | 11.632±0.233 |  | 31 |
|  |  |  |  |  |  |
| 4G, 4H | **SJ4100**  ***zcIs13* (HSP-6::GFP)** | OP50(dead) |  |  |  |
|  |  |  |  |  |  |
|  | EXP.1 | 20℃/control | 7.241±0.071 | <0.0001 | 47 |
|  | EXP.1 | 20℃/50μM CA | 9.124±0.134 |  | 35 |
|  | EXP.2 | 20℃/control | 6.538±0.113 | <0.0001 | 44 |
|  | EXP.2 | 20℃/50μM CA | 9.530±0.135 |  | 32 |
|  | EXP.3 | 20℃/control | 6.292±0.168 | <0.0001 | 33 |
|  | EXP.3 | 20℃/50μM CA | 8.848±0.142 |  | 41 |
|  |  |  |  |  |  |
| 4C,4D | **SJ4005**  ***zcIs4V* (HSP-4::GFP)** | OP50(dead) |  |  |  |
|  |  |  |  |  |  |
|  | EXP.1 | 20℃/control | 7.661±0.450 | <0.0001 | 34 |
|  | EXP.1 | 20℃/50μM CA | 10.256±0.303 |  | 31 |
|  | EXP.2 | 20℃/control | 8.766±0.375 | <0.0001 | 30 |
|  | EXP.2 | 20℃/50μM CA | 12.433±0.354 |  | 36 |

|  | EXP.3 | 20℃/control | 9.934±0.341 | <0.0001 | 43 |
| --- | --- | --- | --- | --- | --- |
|  | EXP.3 | 20℃/50μM CA | 12.383±0.523 |  | 30 |
|  |  |  |  |  |  |
| 4E, 4F | **SJ4058**  ***zcIs13*(HSP-60::GFP)** | OP50(dead) |  |  |  |
|  |  |  |  |  |  |
|  | EXP.1 | 20℃/control | 7.661±0.450 | <0.0001 | 34 |
|  | EXP.1 | 20℃/50μM CA | 10.256±0.303 |  | 31 |
|  | EXP.2 | 20℃/control | 8.766±0.375 | <0.0001 | 30 |
|  | EXP.2 | 20℃/50μM CA | 12.433±0.354 |  | 36 |
|  | EXP.3 | 20℃/control | 9.934±0.341 | <0.0001 | 43 |
|  | EXP.3 | 20℃/50μM CA | 12.383±0.523 |  | 30 |
|  |  |  |  |  |  |

*P* value was determined by comparison between the control group and the experimental group in each independent experiment. The data in the experimental group were counted as the number of normal dead nematodes. The experimental data were processed and analyzed by SPSS26.0 with Kaplan-Meier survival curve. P value was obtained by log-rank test analysis.

N: number of dead nematodes.

**Table S6. Effect of CA on resistance to bacteria, high temperature and oxidation in wild-type nematode (N2).**

| Figure | Strain | Treatment | Mean lifespan  ±SEM  (Days) | P value  VS  control | change in mean lifespan | N |
| --- | --- | --- | --- | --- | --- | --- |
| 3A | **N2** | **Paraquat(dead)** | Day |  |  |  |
|  | EXP.1 | 20℃/control | 6.394±0.335 | 0.02 | 18.52% | 94 |
|  | EXP.1 | 20℃/50μM CA | 7.578±0.343 |  |  | 90 |
|  | EXP.2 | 20℃/control | 6.081±0.353 | 0.041 | 18.93% | 86 |
|  | EXP.2 | 20℃/50μM CA | 7.232±0.365 |  |  | 82 |
|  | EXP.3 | 20℃/control | 6.091±0.303 | 0.008 | 20.97% | 99 |
|  | EXP.3 | 20℃/50μM CA | 7.368±0.303 |  |  | 95 |
|  |  |  |  |  |  |  |
| 4A | **N2** | OP50(dead) | Hours |  |  |  |
|  | EXP.1 | 35℃/control | 10.615±0.394 | <0.001 | 17.94% | 78 |
|  | EXP.1 | 35℃/50μM CA | 12.519±0.355 |  |  | 77 |
|  | EXP.2 | 35℃/control | 10.612±0.278 | <0.001 | 18.89% | 98 |
|  | EXP.2 | 35℃/50μM CA | 12.617±0.449 |  |  | 81 |
|  | EXP.3 | 35℃/control | 10.485±0.301 | <0.001 | 15.71% | 99 |
|  | EXP.3 | 35℃/50μM CA | 12.132±0.308 |  |  | 91 |
|  |  |  |  |  |  |  |

| Figure | Strain | Treatment | Mean±SEM | P value  VS  Control | N |
| --- | --- | --- | --- | --- | --- |
|  | **N2(WT)** | OP50(dead) |  |  |  |
| 3C, 3D | EXP.1 | 20℃/Control | 17.211±0.975 |  | 40 |
|  | EXP.1 | 20℃/50μM CA | 11.160±0.326 | <0.0001 | 33 |
|  | EXP.1 | 20℃/20mM Paraquat | 23.986±1.804 | <0.0001 | 36 |
|  | EXP.1 | 20℃/2mM NAC | 11.450±0.340 | <0.0001 | 42 |
|  |  |  |  |  |  |
|  | EXP.2 | 20℃/Control | 16.308±0.601 |  | 35 |
|  | EXP.2 | 20℃/50μM CA | 12.864±0.360 | <0.0001 | 39 |
|  | EXP.2 | 20℃/20mM Paraquat | 27.940±2.300 | <0.0001 | 33 |
|  | EXP.2 | 20℃/2mM NAC | 11.572±0.667 | <0.0001 | 35 |
|  |  |  |  |  |  |
|  | EXP.3 | 20℃/Control | 20.727±0.196 |  | 30 |
|  | EXP.3 | 20℃/50μM CA | 14.449±0.519 | <0.0001 | 38 |
|  | EXP.3 | 20℃/20mM Paraquat | 41.199±1.148 | <0.0001 | 30 |
|  | EXP.3 | 20℃/2mM NAC | 13.612±.618 | <0.0001 | 33 |
|  |  |  |  |  |  |

**Table S7. Effect of CA on ROS level of N2 nematode.**

**Table S8. Effects of CA on age-related diseases.**

| **PD** |  |  |  |  |  |
| --- | --- | --- | --- | --- | --- |
| Figure | Strain | Treatment | Mean±SEM  Days | P value  VS  6-OH DA | N |
|  | **BZ555**  ***egIs1*(DAT-1::GFP)** | OP50(dead) |  |  |  |
| 2E, 2F | EXP.1 | 20℃/50mM 6-OHDA | 7.549±0.281 |  | 28 |
|  | EXP.1 | 20℃/50mM 6-OHDA  +50μM CA | 13.792±0.814 | <0.0001 | 23 |
|  | EXP.1 | 20℃/Control | 16.962±1.292 | <0.0001 | 23 |
|  | EXP.1 | 20℃/50mM 6-OHDA  +2mM L-DA | 15.752±1.018 | <0.0001 | 20 |
|  | EXP.2 | 20℃/50mM 6-OHDA | 8.014±0.429 |  | 23 |
|  | EXP.2 | 20℃/50mM 6-OHDA  +50μM CA | 13.731±0.869 | <0.0001 | 22 |
|  | EXP.2 | 20℃/Control | 16.986±1.569 | <0.0001 | 20 |
|  | EXP.2 | 20℃/50mM 6-OHDA  +2mM L-DA | 15.180±1.178 | <0.0001 | 21 |
|  | EXP.3 | 20℃/6-OHDA | 6.072±0.386 |  | 22 |
|  | EXP.3 | 20℃/50mM 6-OHDA  +50μM CA | 14.543±1.020 | <0.0001 | 24 |
|  | EXP.3 | 20℃/Control | 14.438±1.148 | <0.0001 | 20 |
|  | EXP.3 | 20℃/50mM 6-OHDA  +2mM L-DA | 13.922±.978 | <0.0001 | 21 |
|  |  |  |  |  |  |
| 2C, 2D | **N5901**  **Punc-54::α-syn::YFP** | OP50(dead) |  |  |  |
|  |  |  |  |  |  |
|  | EXP.1 | 20℃/control | 22.344±0.475 | <0.0001 | 33 |
|  | EXP.1 | 20℃/50μM CA | 10.172±0.272 |  | 37 |
|  | EXP.2 | 20℃/control | 20.436±0.465 | <0.0001 | 35 |
|  | EXP.2 | 20℃/50μM CA | 10.650±0.407 |  | 30 |
|  | EXP.3 | 20℃/control | 21.678±0.580 | <0.0001 | 31 |
|  | EXP.3 | 20℃/50μM CA | 11.391±0.447 |  | 31 |
|  |  |  |  |  |  |

| AD |  |  |  |  |  |  |
| --- | --- | --- | --- | --- | --- | --- |
| Figure | Strain | Treatment | Mean lifespan  ±SEM  (hours) | P value  VS  control | change in mean lifespan | N |
| 2A | **CL2006**  **(A-beta)** | OP50(dead) | Days |  |  |  |
|  | EXP.1 | 20℃/control | 16.569±0.719 | <0.001 | 16.18% | 65 |
|  | EXP.1 | 20℃/50μM CA | 19.250±0.746 |  |  | 64 |
|  | EXP.2 | 20℃/control | 16.543±0.590 | 0.013 | 14.68% | 81 |
|  | EXP.2 | 20℃/50μM CA | 18.972±0.525 |  |  | 71 |
|  | EXP.3 | 20℃/control | 16.282±0.512 | <0.001 | 17.62% | 110 |
|  | EXP.3 | 20℃/50μM CA | 19.152±0.494 |  |  | 102 |
|  |  |  |  |  |  |  |

**Table S9. Effect of CA on oviposition rate of N2 worm.**

| Figure | Days | Strains | Treatments | Mean offspring  ±SEM | P value  VS  control | N |
| --- | --- | --- | --- | --- | --- | --- |
| 7D | 1 | **N2(WT)** | OP50(dead) |  |  |  |
|  |  | EXP.1 | 20℃/control | 33.069±1.566 | 0.186 | 30 |
|  |  | EXP.1 | 20℃/50μM CA | 29.552±2.053 |  |  |
|  | 2 | EXP.1 | 20℃/control | 78.655±5.183 | 0.509 | 30 |
|  |  | EXP.1 | 20℃/50μM CA | 73.310±5.974 |  |  |
|  | 3 | EXP.1 | 20℃/control | 51.414±2.940 | 0.31 | 30 |
|  |  | EXP.1 | 20℃/50μM CA | 47.034±3.005 |  |  |
|  | 4 | EXP.1 | 20℃/control | 26.586±1.798 | 0.015 | 30 |
|  |  | EXP.1 | 20℃/50μM CA | 20.448±1.588 |  |  |
|  | 5 | EXP.1 | 20℃/control | 28.207±1.443 | 0.932 | 30 |
|  |  | EXP.1 | 20℃/50μM CA | 28.483±2.837 |  |  |
|  | 6 | EXP.1 | 20℃/control | 15.724±0.999 | 0.008 | 30 |
|  |  | EXP.1 | 20℃/50μM CA | 20.552±1.422 |  |  |
|  | 7 | EXP.1 | 20℃/control | 2.931±0.548 | 0.121 | 30 |
|  |  | EXP.1 | 20℃/50μM CA | 4.276±0.638 |  |  |
|  | Total | EXP.1 | 20℃/control | 236.586±6.020 | 0.169 | 30 |
|  |  | EXP.1 | 20℃/50μM CA | 223.655±6.868 |  |  |
|  |  |  |  |  |  |  |
|  |  | **N2（WT）** | OP50(dead) |  |  |  |
|  | 1 | EXP.2 | 20℃/control | 28.767±1.762 | 0.155 | 29 |
|  |  | EXP.2 | 20℃/50μM CA | 31.900±1.335 |  |  |
|  | 2 | EXP.2 | 20℃/control | 75.333±3.389 | 0.857 | 29 |
|  |  | EXP.2 | 20℃/50μM CA | 76.533±5.828 |  |  |
|  | 3 | EXP.2 | 20℃/control | 57.333±2.900 | 0 | 29 |
|  |  | EXP.2 | 20℃/50μM CA | 41.600±3.042 |  | 29 |
|  | 4 | EXP.2 | 20℃/control | 22.967±1.963 | 0.075 | 29 |
|  |  | EXP.2 | 20℃/50μM CA | 27.567±1.764 |  |  |
|  | 5 | EXP.2 | 20℃/control | 25.933±1.307 | 0.856 | 29 |
|  |  | EXP.2 | 20℃/50μM CA | 25.533±1.801 |  |  |
|  | 6 | EXP.2 | 20℃/control | 11.033±1.007 | 0 | 29 |
|  |  | EXP.2 | 20℃/50μM CA | 20.600±1.206 |  |  |
|  | 7 | EXP.2 | 20℃/control | 1.767±0.518 | 0.232 | 29 |
|  |  | EXP.2 | 20℃/50μM CA | 226.500±7.660 |  |  |
|  | Total | EXP.2 | 20℃/control | 223.033±6.802 | 0.732 | 29 |
|  |  | EXP.2 | 20℃/50μM CA | 226.500±7.660 |  |  |
|  |  |  |  |  |  |  |
|  |  | **N2（WT）** | OP50(dead) |  |  |  |
|  | 1 | EXP.3 | 20℃/control | 23.167±1.393 | 0.623 | 30 |
|  |  | EXP.3 | 20℃/50μM CA | 24.100±1.274 |  |  |
|  | 2 | EXP.3 | 20℃/control | 65.000±3.265 | 0.304 | 30 |
|  |  | EXP.3 | 20℃/50μM CA | 69.533±2.911 |  |  |
|  | 3 | EXP.3 | 20℃/control | 67.933±3.054 | 0.908 | 30 |
|  |  | EXP.3 | 20℃/50μM CA | 67.333±4.178 |  |  |
|  | 4 | EXP.3 | 20℃/control | 25.733±2.100 | 0.732 | 30 |
|  |  | EXP.3 | 20℃/50μM CA | 26.600±1.384 |  |  |
|  | 5 | EXP.3 | 20℃/control | 26.000±1.490 | 0.007 | 30 |
|  |  | EXP.3 | 20℃/50μM CA | 20.333±1.379 |  |  |
|  | 6 | EXP.3 | 20℃/control | 10.633±1.280 | 0.683 | 30 |
|  |  | EXP.3 | 20℃/50μM CA | 9.967±1.003 |  |  |
|  | 7 | EXP.3 | 20℃/control | 4.900±0.866 | 0.454 | 30 |
|  |  | EXP.3 | 20℃/50μM CA | 4.033±0.756 |  |  |
|  | Total | EXP.3 | 20℃/control | 223.367±6.915 | 0.878 | 29 |
|  |  | EXP.3 | 20℃/50μM CA | 221.900±6.559 |  |  |
|  |  |  |  |  |  |  |

**Table S10. Effect of CA on gene expression at mRNA level in nematode.**

| **Gene** | | **EXP.1** | | | **EXP.2** | | **EXP.3** | | | | **Mean** | | | **SEM** | |  |  |  |  |
| --- | --- | --- | --- | --- | --- | --- | --- | --- | --- | --- | --- | --- | --- | --- | --- | --- | --- | --- | --- |
| Control | | 1 | | | 1 | | 1 | | | | 1 | | | 0 | |  |  |  |  |
| *sod-3* | | 2.17 | | | 1.473 | | 1.463 | | | | 1.602 | | | 0.243 | |  |  |  |  |
| *gst-4* | | 1.612 | | | 1.789 | | 1.957 | | | | 1.969 | | | 0.14 | |  |  |  |  |
| *skn-1* | | 0.947 | | | 1.750 | | 1.417 | | | | 1.687 | | | 0.115 | |  |  |  |  |
| *hsf-1* | | 1.75 | | | 1.778 | | 1.81 | | | | 1.779 | | | 0.148 | |  |  |  |  |
| *dod-3* | | 4.205 | | | 4.176 | | 6.61 | | | | 4.997 | | | 0.659 | |  |  |  |  |
| *ctl-3* | | 1.214 | | | 1.269 | | 1.241 | | | | 1.241 | | | 0.013 | |  |  |  |  |
| *hsp-60* | | 1.262 | | | 1.368 | | 1.261 | | | | 1.264 | | | 0.049 | |  |  |  |  |
| *hsp-16.2* | | 1.608 | | | 1.754 | | 1.68 | | | | 1.681 | | | 0.034 | |  |  |  |  |
| *hsp-16.1* | | 4.902 | | | 4.491 | | 5.250 | | | | 4.881 | | | 0.275 | |  |  |  |  |
|  | | Control | | | | | 50 μM CA | | | | *daf-16(mu86)I* | | | | | *daf-16(mu86)I.* 50μM CA | | | |
| *sod-3* | | 1.000 | | 1.000 | 1.000 | | 2.170 | | 1.473 | 1.463 | 0.679 | | 0.682 | 0.594 | | 0.718 | | 0.686 | 0.668 |
| *sod-2* | | 1.000 | | 1.000 | 1.000 | | 1.618 | | 1.594 | 1.570 | 0.981 | | 0.930 | 0.732 | | 0.893 | | 1.192 | 0.818 |
| *daf-16* | | 1.000 | | 1.000 | 1.000 | | 1.508 | | 1.404 | 1.307 | 0.807 | | 0.827 | 0.831 | | 1.249 | | 1.278 | 1.003 |
| *dod-3* | | 1.000 | | 1.000 | 1.000 | | 4.205 | | 4.176 | 6.610 | 0.822 | | 0.754 | 0.925 | | 0.897 | | 1.122 | 0.815 |
| *F22B5.4* | | 1.000 | | 1.000 | 1.000 | | 2.091 | | 2.473 | 2.068 | 0.625 | | 0.866 | 0.848 | | 0.991 | | 0.903 | 0.679 |
| *ctl-3* | | 1.000 | | 1.000 | 1.000 | | 1.214 | | 1.269 | 1.241 | 0.850 | | 0.996 | 0.836 | | 0.871 | | 1.039 | 0.951 |
|  | |  | |  |  | |  | |  |  |  | |  |  | |  | |  |  |
|  | | Control | | | | | 50 μM CA | | | | *skn-1 (zu67)IV* | | | | | *skn-1(zu67)IV.* 50 μM CA | | | |
| *skn-1* | | 1.000 | | 1.000 | 1.000 | | 0.947 | | 1.750 | 1.417 | 0.666 | | 0.624 | 0.745 | | 0.730 | | 0.758 | 0.854 |
| *gcs-1* | | 1.000 | | 1.000 | 1.000 | | 1.661 | | 1.988 | 2.257 | 0.929 | | 0.961 | 0.891 | | 0.926 | | 1.256 | 1.206 |
| *gst-4* | | 1.000 | | 1.000 | 1.000 | | 1.612 | | 1.789 | 1.957 | 0.635 | | 0.618 | 0.602 | | 0.661 | | 0.809 | 0.890 |
| *nhr-57* | | 1.000 | | 1.000 | 1.000 | | 1.629 | | 1.590 | 1.481 | 0.819 | | 0.838 | 0.929 | | 0.843 | | 0.900 | 0.814 |
|  | |  | |  |  | |  | |  |  |  | |  |  | |  | |  |  |
|  | | Control | | | | | 50 μM CA | | | | *hsf-1(sy441)I* | | | | | *hsf-1(sy441)I.* 50 μM CA | | | |
| *hsf-1* | | 1.000 | | 1.000 | 1.000 | | 1.750 | | 1.778 | 1.810 | 0.893 | | 0.909 | 0.850 | | 0.840 | | 1.034 | 0.802 |
| *hsp-60* | | 1.000 | | 1.000 | 1.000 | | 1.262 | | 1.368 | 1.261 | 0.744 | | 0.756 | 0.768 | | 0.763 | | 0.872 | 0.909 |
| *hsp-16.1* | | 1.000 | | 1.000 | 1.000 | | 4.902 | | 4.491 | 5.250 | 0.821 | | 0.917 | 1.118 | | 0.928 | | 1.009 | 0.983 |
| *hsp-16.2* | | 1.000 | | 1.000 | 1.000 | | 1.608 | | 1.754 | 1.68 | 0.712 | | 0.673 | 0.753 | | 0.665 | | 0.756 | 1.158 |
| *dve-1* | | 1.000 | | 1.000 | 1.000 | | 1.618 | | 1.594 | 1.570 | 0.928 | | 0.955 | 1.110 | | 0.926 | | 1.256 | 1.206 |
|  | |  | |  |  | |  | |  |  |  | |  |  | |  | |  |  |

**Table S11. Primers used for the analysis of mRNA expression levels in nematode.**

| gene | Forward primer | Reverse primer |
| --- | --- | --- |
| *cdc-42* | CTGCTGGACAGGAAGATTACG | CTCGGACATTCTCGAATGAAG |
| *F35E12.5* | ACACAATCATTTGCGATGGA | GGTAGTCATTGGAGCCGAAA |
| *T24B8.5* | AAACCTGTGGTGTCTGCGTTAC | TGGCAGGTTTTTGGGCATTG |
| *F08G5.6* | ATCGTTCCGAATGGTGGTTGAC | GCCGATTTCAGCTTGCAAAGTG |
| *F55G11.4* | GGATCCGTGTATTTGGCTGGAATCG | GTGAAGACATATGTGCTCCCGCGTT |
| *Y22F5A.5* | TGCTGATTTCCGTGCTTTCG | TTCCAACAGCATACACGTCACG |
| *irg-1* | AAGCAGCATGCGTATTTTCA | GCAGCTTCTCCTTTTTCTCC |
| *hsp-16.2* | CTGCAGAATCTCTCCATCTGAGTC | AGATTCGAAGCAACTGCACC |
| *hsp-16.1* | GTCACTTTACCACTATTTCCGTCCAGCTCAACGTTC | CAACGGGCGCTTGCTGAATTGGAATAGATCTTCC |
| *hsp-60* | AGGAGAAGCTTAATGAGCG | ACACGGTCCTTCTTCTCT |
| *hsp-12.6* | GTGATGGCTGACGAAGGAAC | GGGAGGAAGTTATGGGCTTC |
| *dve-1* | TCGAGGCCTCATACAAGAA | AAGAGGTTTTCCACAGTGTC |
| *hsp-6* | AGGAACAACAGAGTAAGATTTTC | TCGATTTGGTCCTTGGAAAG |
| *sod-3* | AGCATCATGCCACCTACGTGA | CACCACCATTGAATTTCAGCG |
| *nhr-57* | GACTCTGTGTGGAGTGATGGAGAG | GTGGCTCTTGGTGTCAATTTCGGG |
| *fard-1* | GGGTTTTTGGGAAAGGTGAT | CCACCGATTGCTTTCAATTT |
| *skn-1* | AGTGTCGGCGTTCCAGATTTC | GTCGACGAATCTTGCGAATCA |
| *daf-12* | AGGCGTTTCGTCAAAGTTGC | CCTGCTCTCCGAACAACGAT |
| *daf-9* | GAGGGCATTCTCCGCAAGT | CCACTGCTGAAGTCGAAATCC |
| *dod-3* | AAGCCATGTTCCCGAATGAG | GCTGCGAAAAGCAAGAAAATG |
| *F22B5.4* | GAGATCCACGTTTTGTTAAAGTCGC | CGGCGGACAAGGAATTGATAAGGAG |
| *daf-16* | CCAGACGGAAGGCTTAAAACT | ATTCGCATGAAACGAGAATG |
| *ctl-1* | GAATGTGAAGAATTATTTCGCTGA | AACTCGATTCCTGGGACGAT |
| *ctl-2* | CAAGGAACTACTTCGCTGAGG | AATGAGTGTCGGTGTACGAGAA |
| *ctl-3* | AGTAAATCTTCAAAATGCCAATG | GGTGGGGTTCCTGATTTCTAT |
| *sod-2* | GATACTGTCCAAAGGGAAAGAT | GTAGTAAGCGTGCTCCCAGA |
| *daf-2* | CGGTGCGAAGAGAGGATATT | TACAGAGGTCGCCGTTACTG |
| *gst-4* | TCCGTCAATTCACTTCTTCCG | AAGAAATCATCACGGGCTGG |
| *hsf-1* | TTGACGACGACAAGCTTCCAGT | AAAGCTTGCACCAGAATCATCCC |
